# Supplementary figures and images for: Synthesis and biological assessment of chalcone and pyrazoline derivatives as novel inhibitor for ELF3-MED23 interaction
Source: eLife. 2024 Dec 5;13:RP97051. doi: 10.7554/eLife.97051 (PMC11623927; doi:10.7554/eLife.97051)

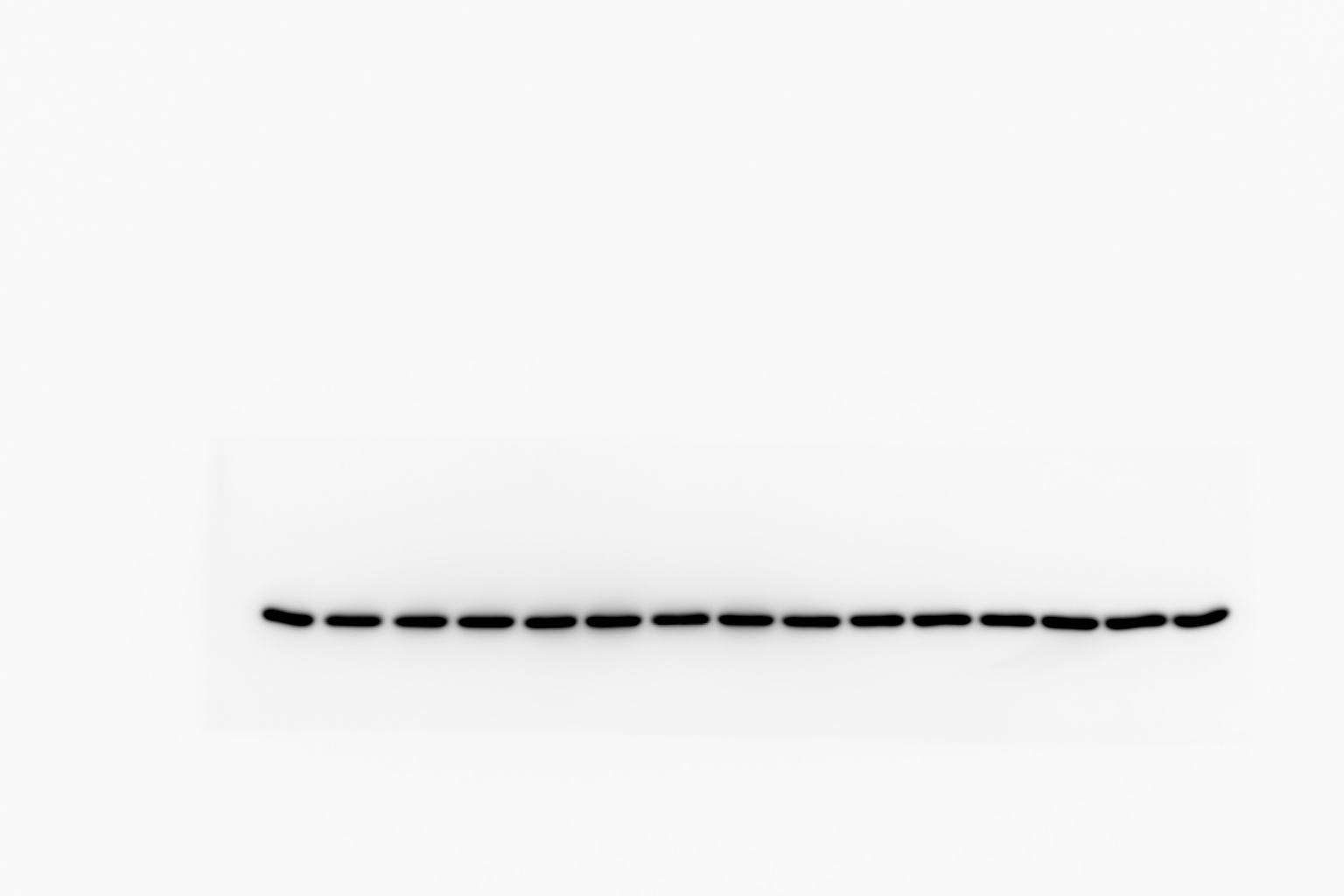

Supplement: Figure 3—source data 1. [file elife-97051-fig3-data1.zip › Figure1C-source data 1. Raw unedited gels for Figure 1C/GAPDH.tif]

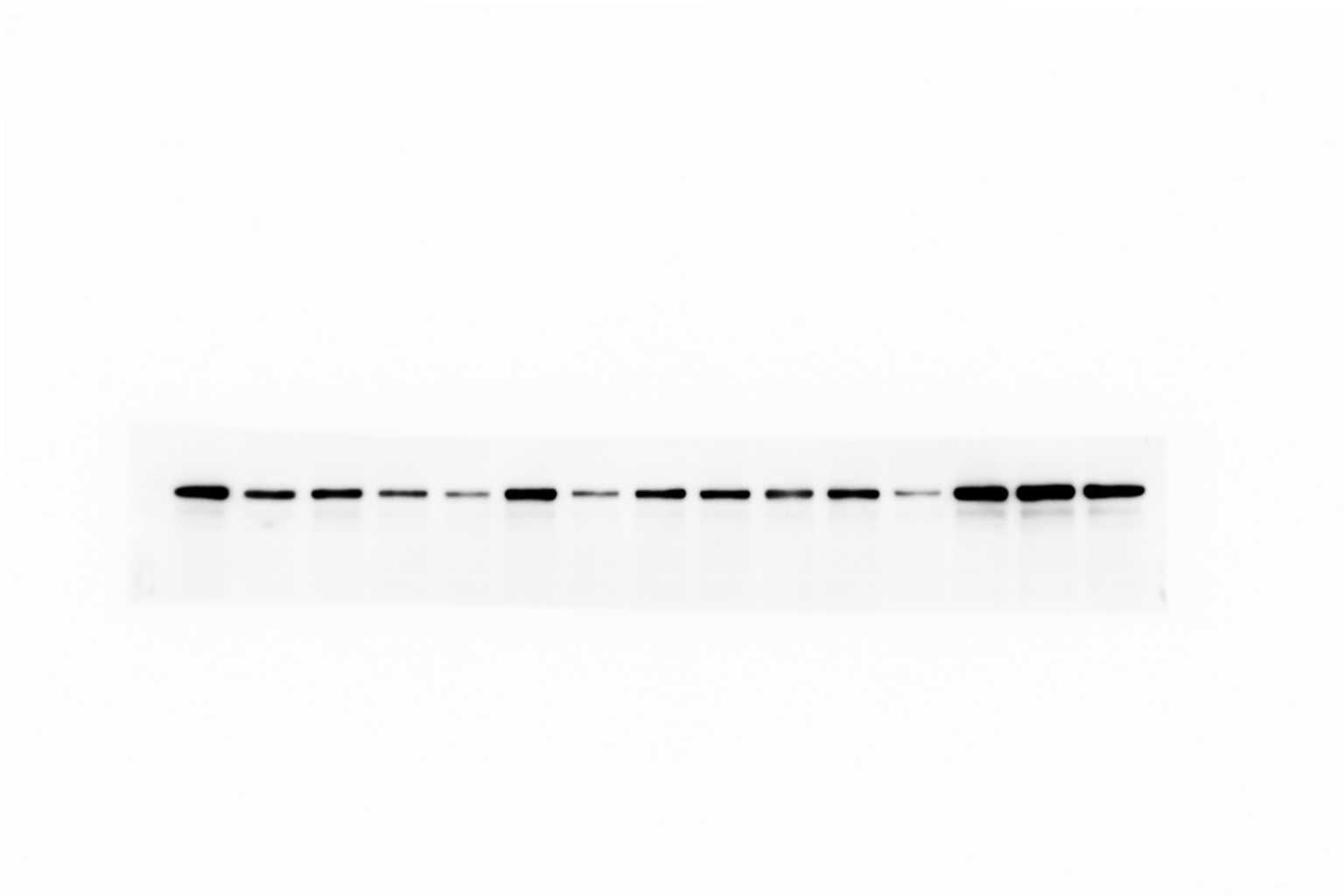

Supplement: Figure 3—source data 1. [file elife-97051-fig3-data1.zip › Figure1C-source data 1. Raw unedited gels for Figure 1C/HER2.tif]

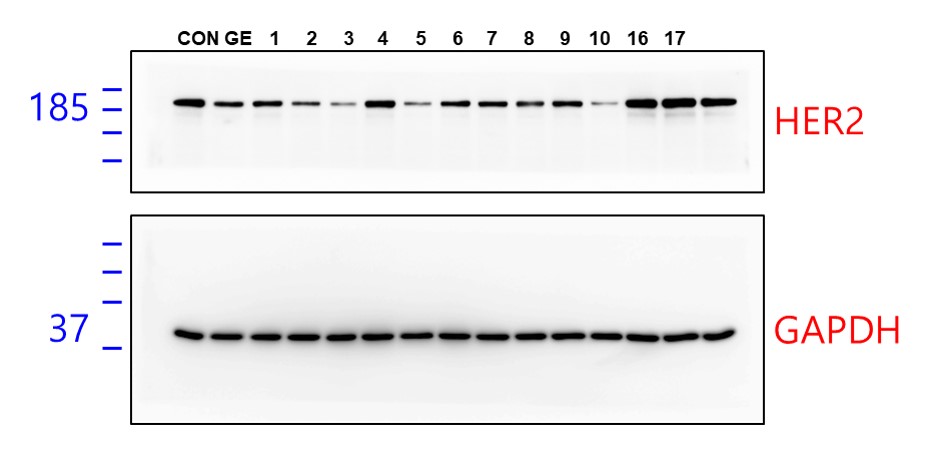

Supplement: Figure 3—source data 2. [file elife-97051-fig3-data2.zip › Figure1C_1.jpg]

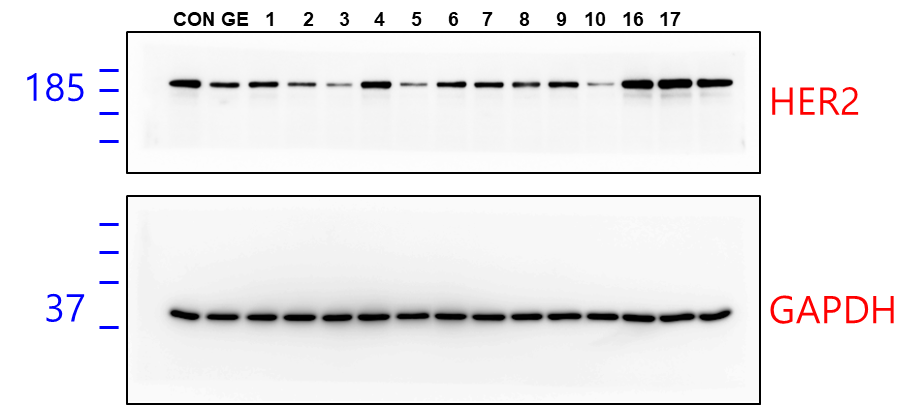

Supplement: Figure 3—source data 2. [file elife-97051-fig3-data2.zip › Figure1C.tif]

## Source Data 2

Uncropped blot images of Figure 3C

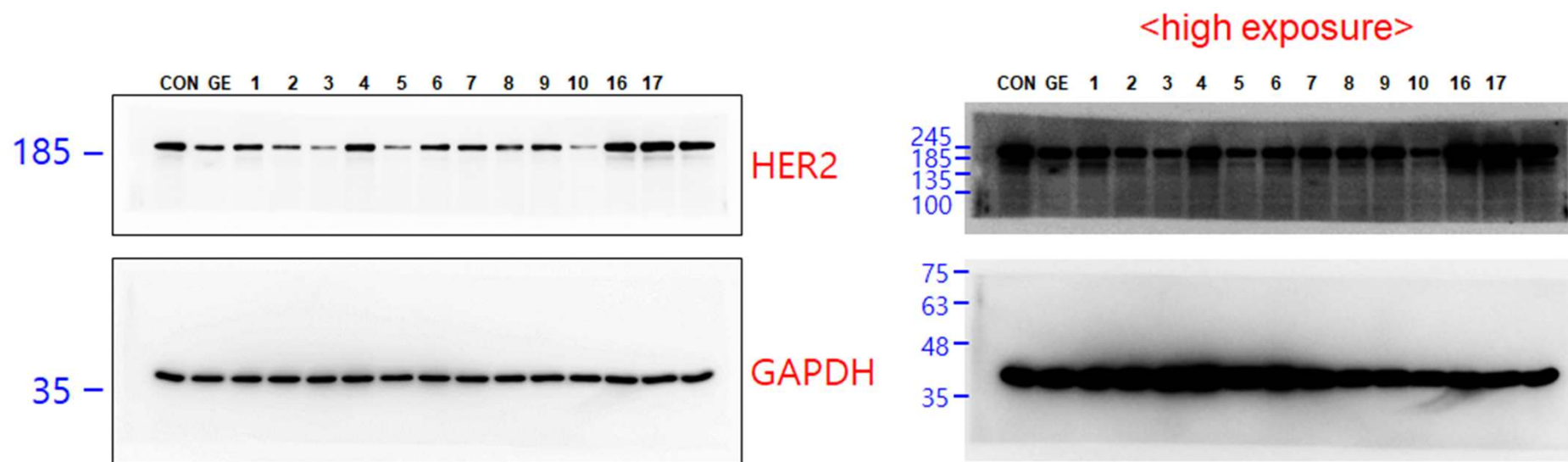

Supplement: Figure 3—source data 2. [file elife-97051-fig3-data2.zip › Source data with molecular weight marker_Part1.pdf]

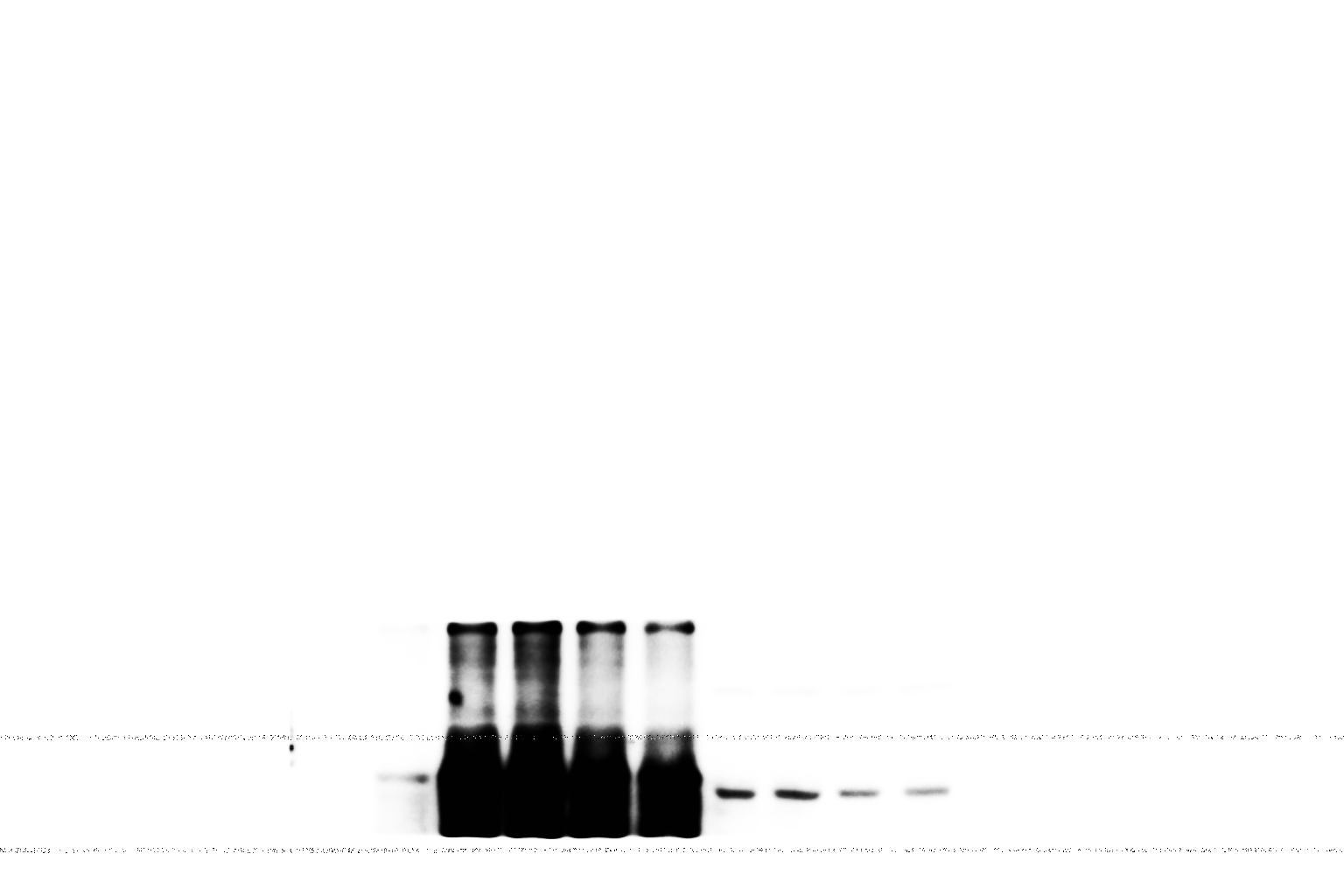

Supplement: Figure 3—source data 3. [file elife-97051-fig3-data3.zip › Figure1E-source data 1. Raw unedited gels for Figure 1E/ELF3_high exp.tif]

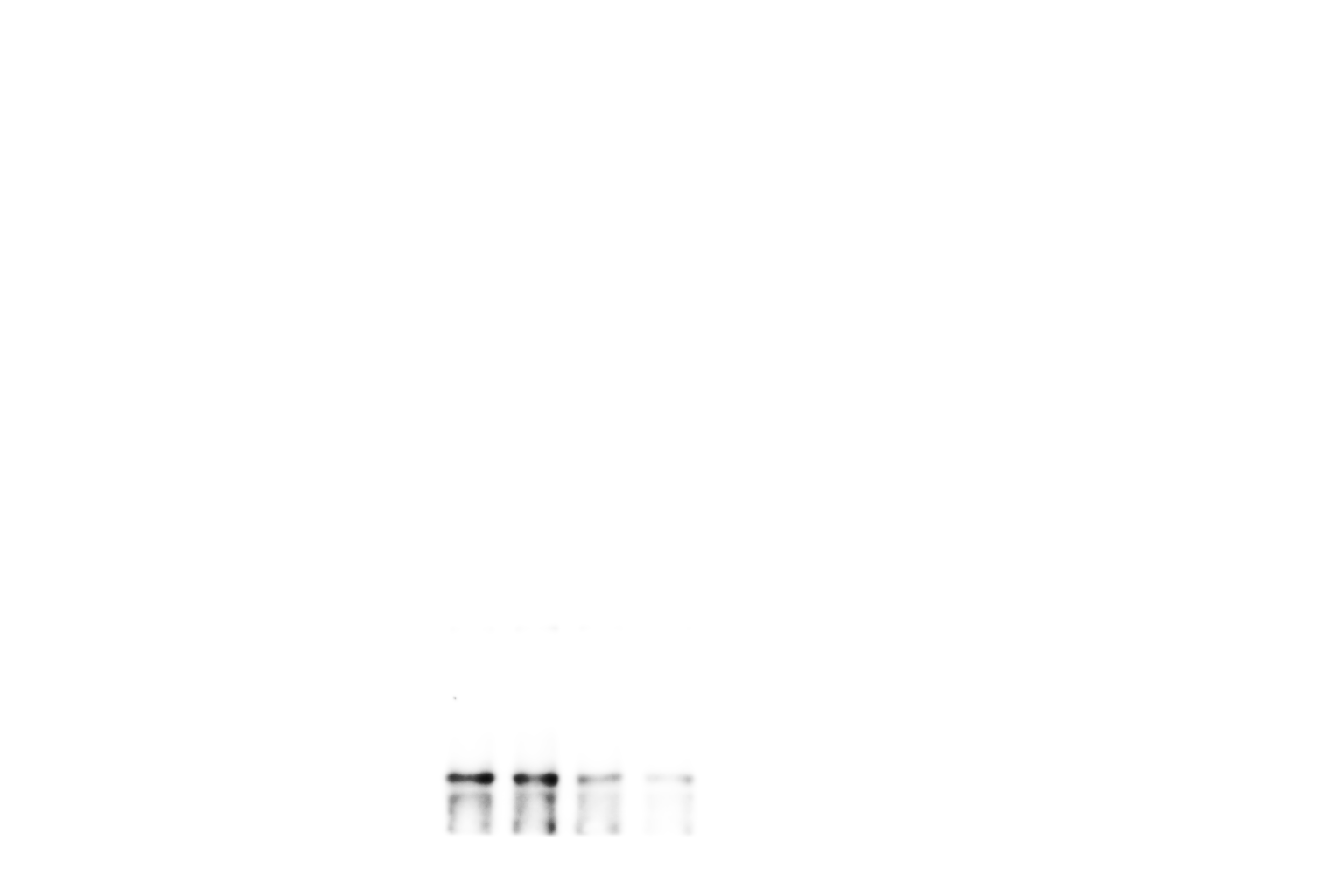

Supplement: Figure 3—source data 3. [file elife-97051-fig3-data3.zip › Figure1E-source data 1. Raw unedited gels for Figure 1E/ELF3_low exp.tif]

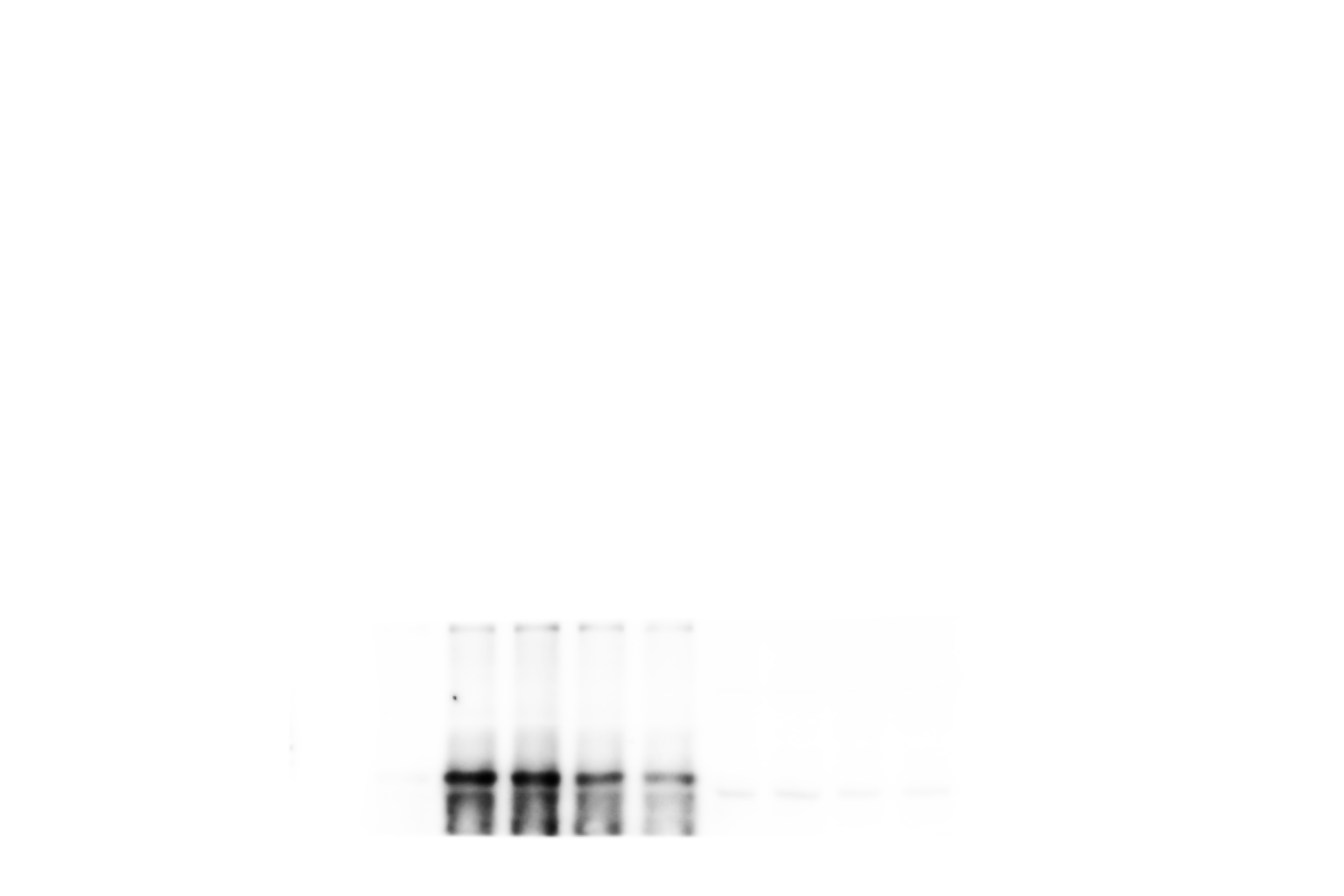

Supplement: Figure 3—source data 3. [file elife-97051-fig3-data3.zip › Figure1E-source data 1. Raw unedited gels for Figure 1E/ELF3_whole.tif]

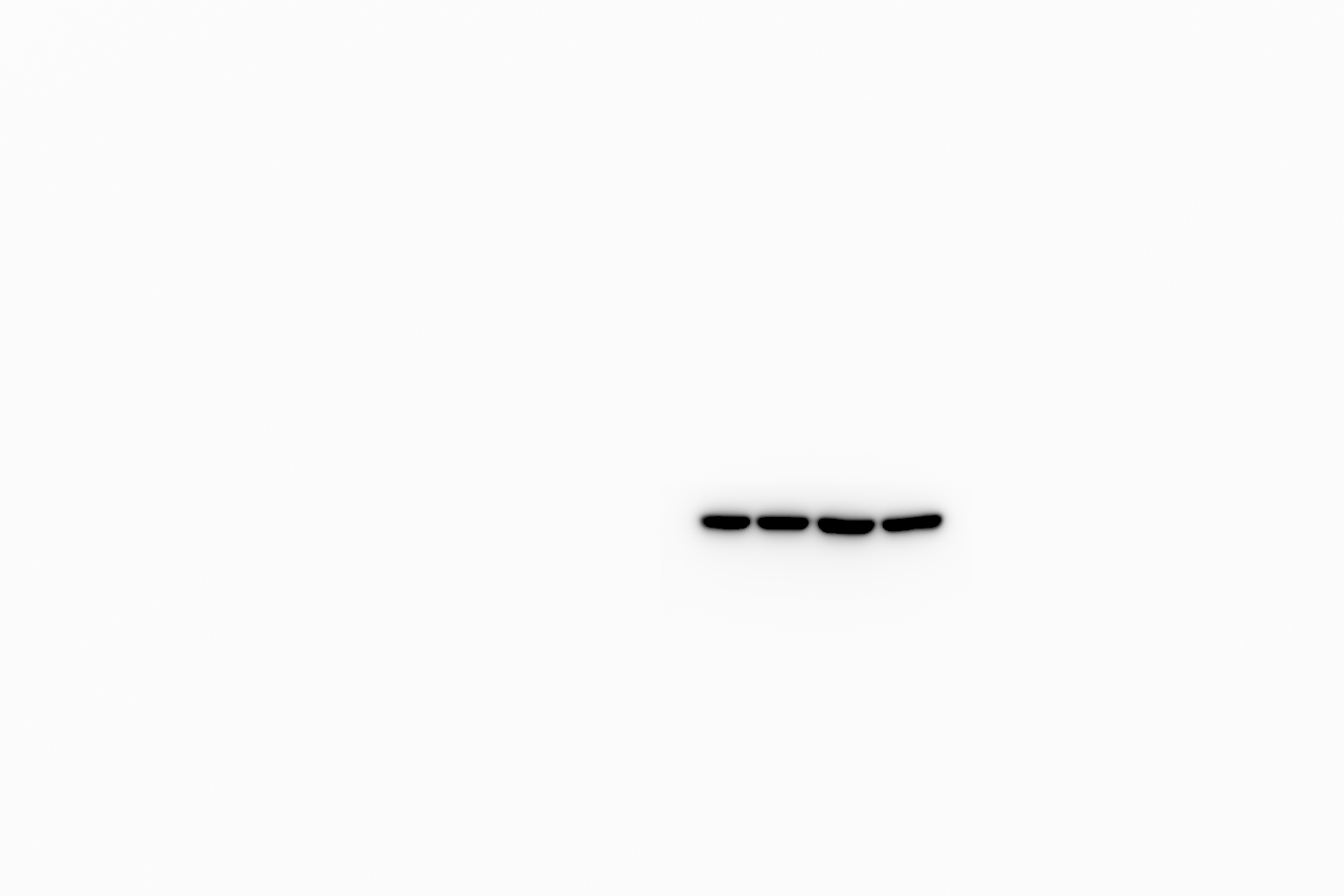

Supplement: Figure 3—source data 3. [file elife-97051-fig3-data3.zip › Figure1E-source data 1. Raw unedited gels for Figure 1E/GAPDH.tif]

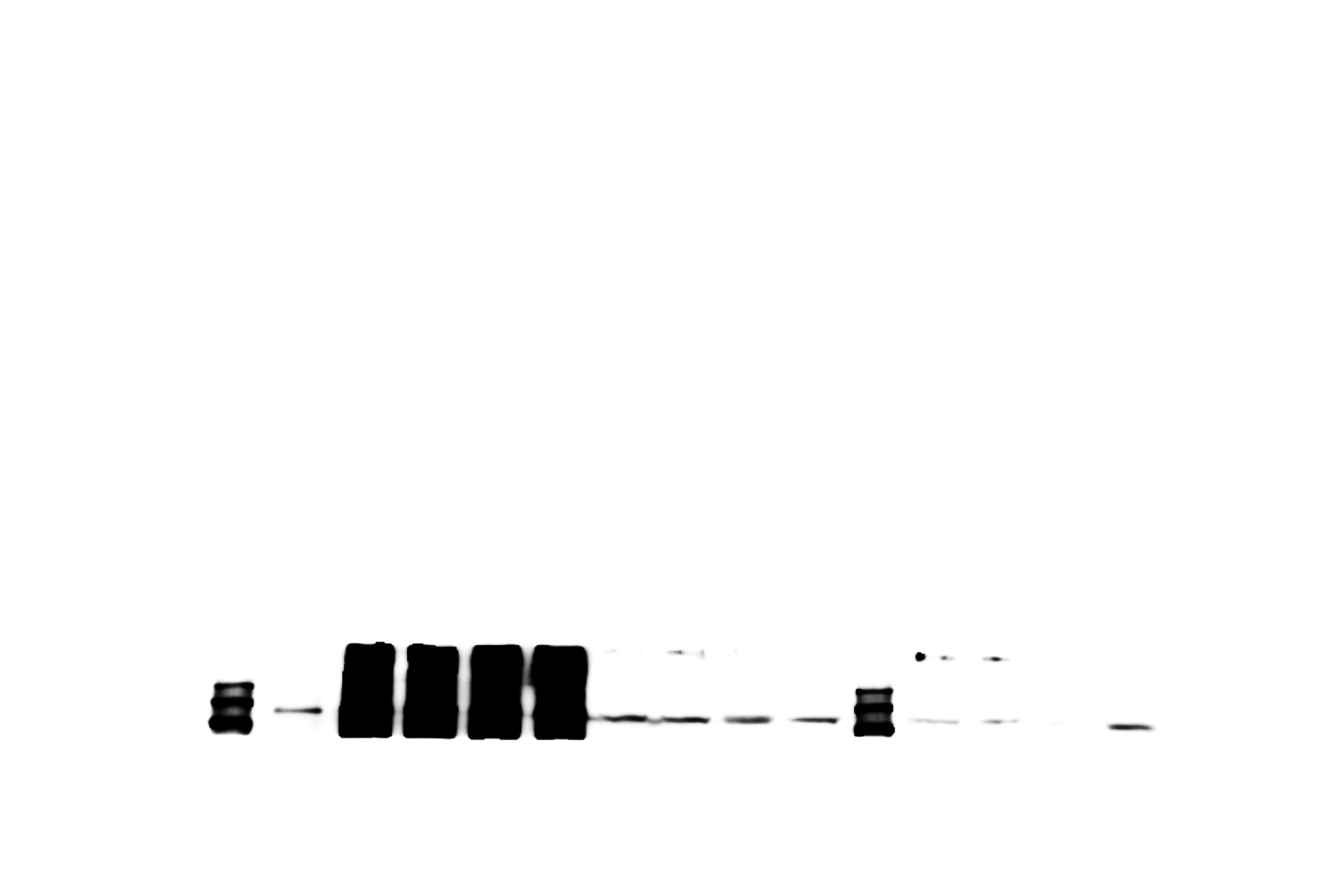

Supplement: Figure 3—source data 3. [file elife-97051-fig3-data3.zip › Figure1E-source data 1. Raw unedited gels for Figure 1E/MED23_high exp.tif]

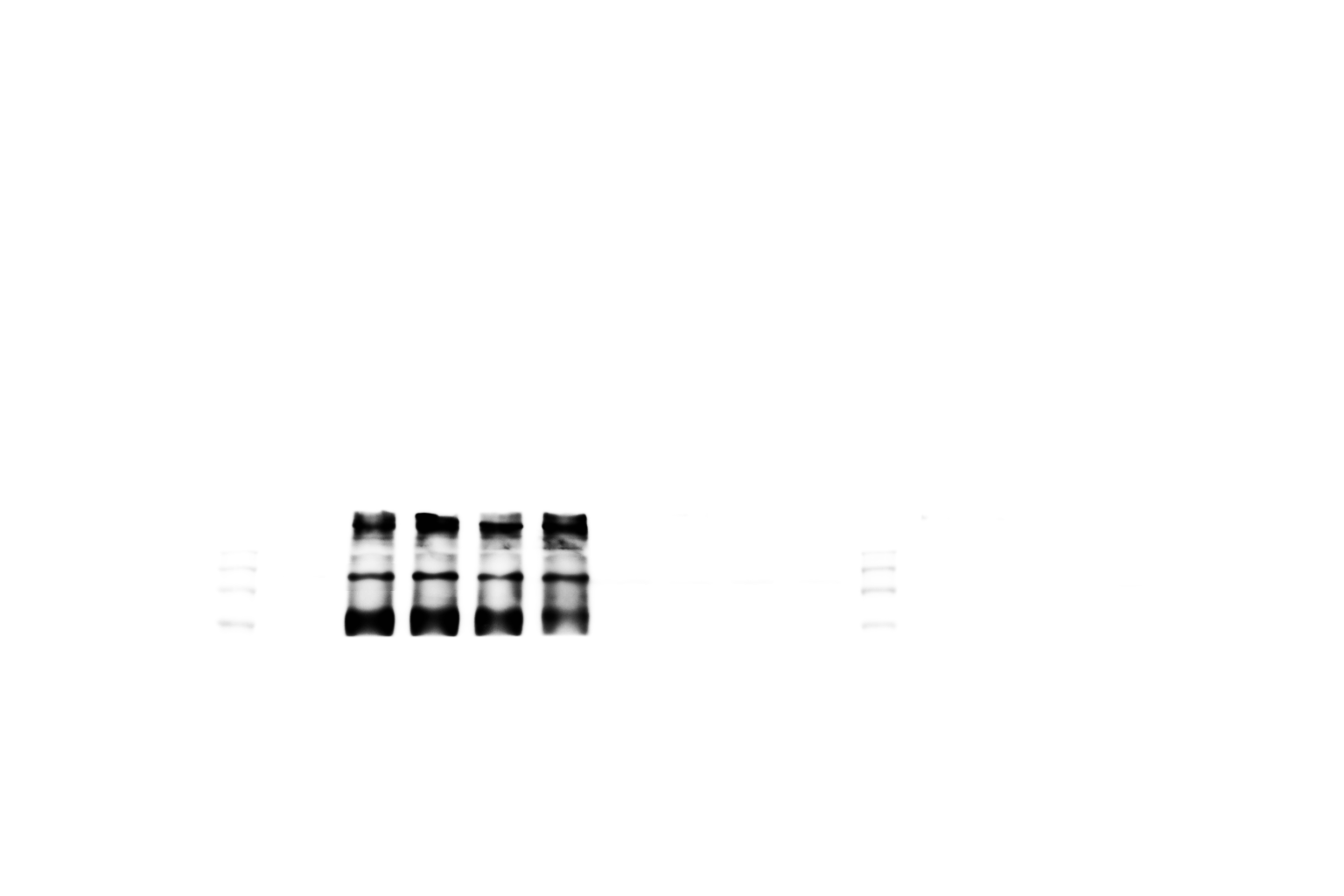

Supplement: Figure 3—source data 3. [file elife-97051-fig3-data3.zip › Figure1E-source data 1. Raw unedited gels for Figure 1E/MED23_low exp.tif]

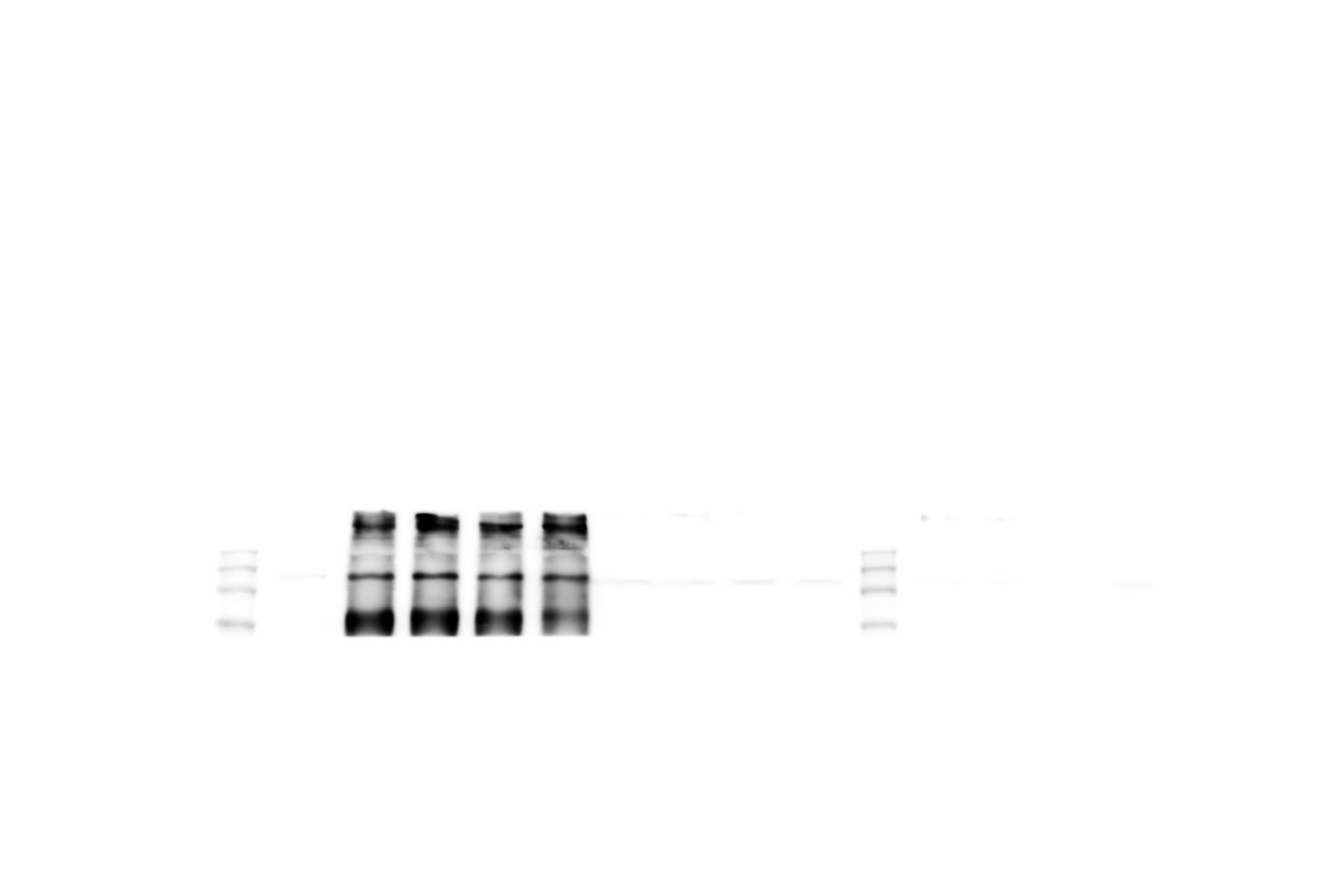

Supplement: Figure 3—source data 3. [file elife-97051-fig3-data3.zip › Figure1E-source data 1. Raw unedited gels for Figure 1E/MED23_whole.tif]

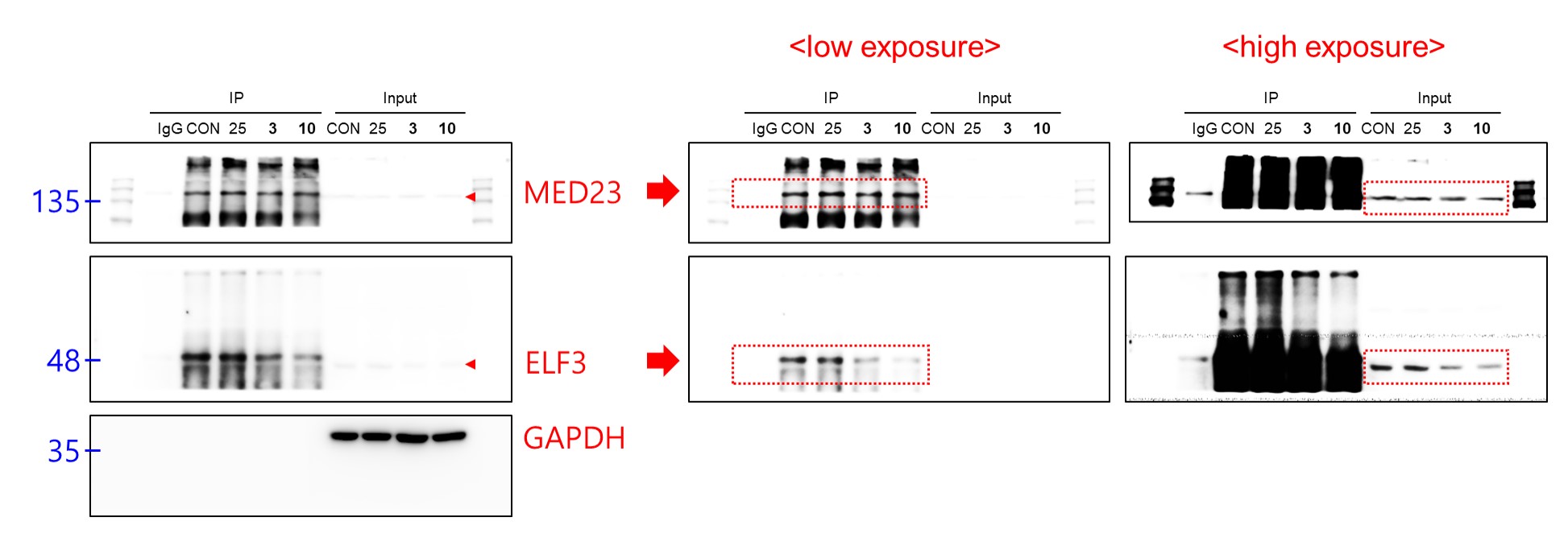

Supplement: Figure 3—source data 4. [file elife-97051-fig3-data4.zip › Figure1E_1.jpg]

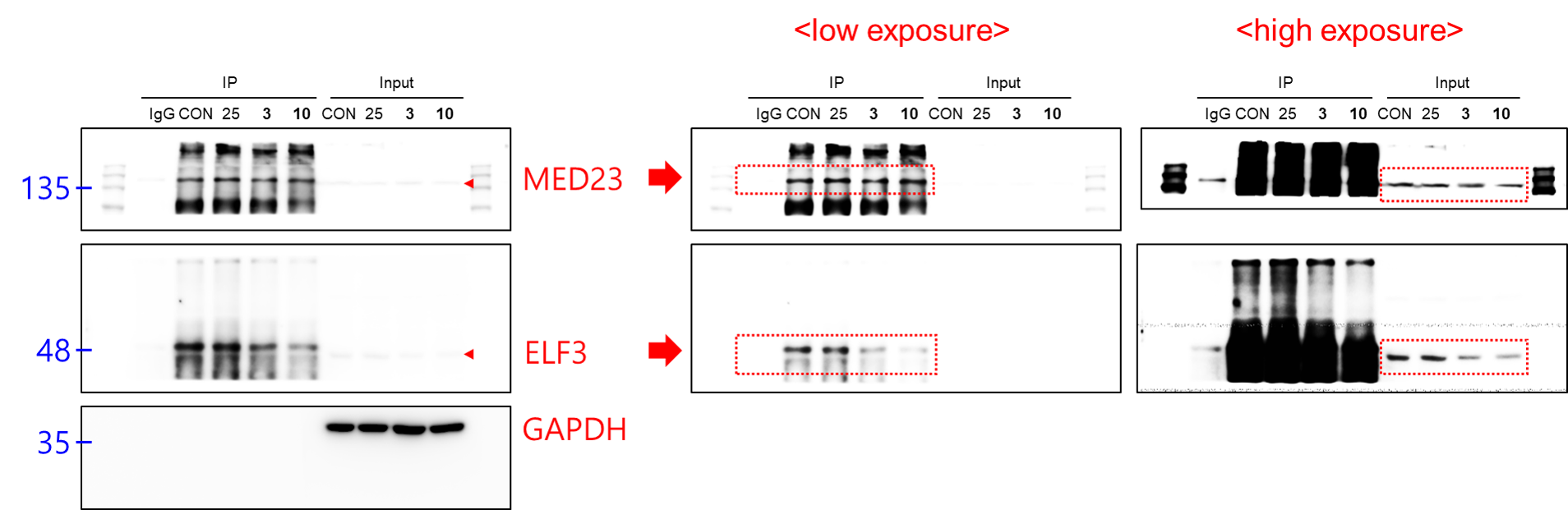

Supplement: Figure 3—source data 4. [file elife-97051-fig3-data4.zip › Figure1E.tif]

## Source Data 2

### Uncropped blot images of Figure 3E

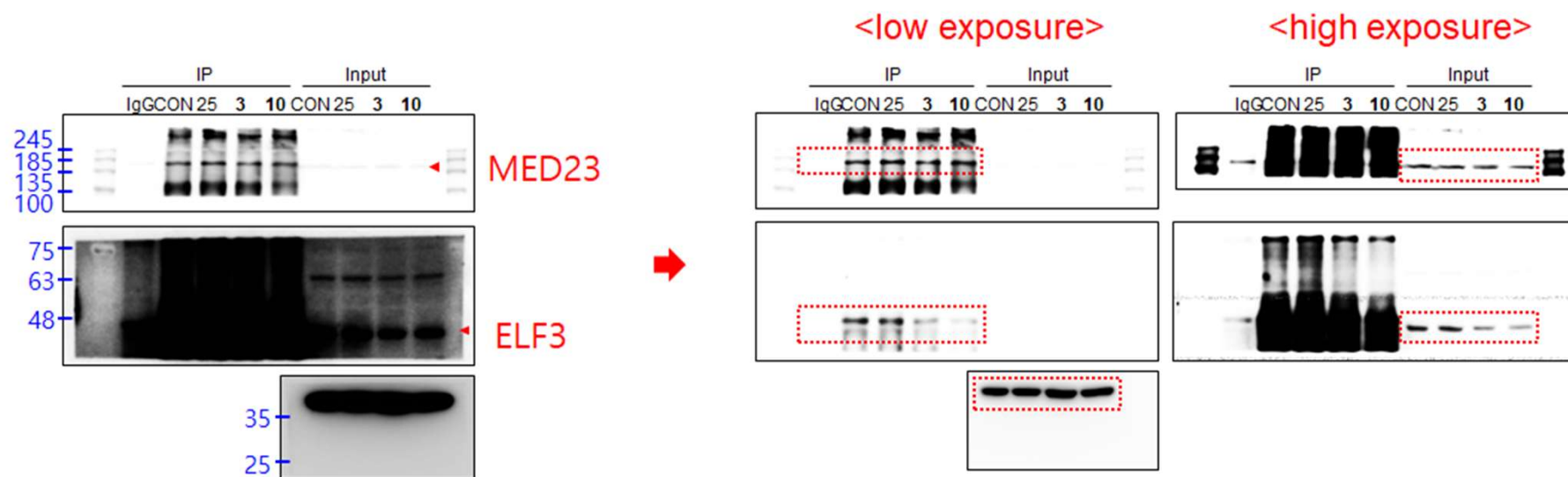

Supplement: Figure 3—source data 4. [file elife-97051-fig3-data4.zip › Source data with molecular weight marker_Part2.pdf]

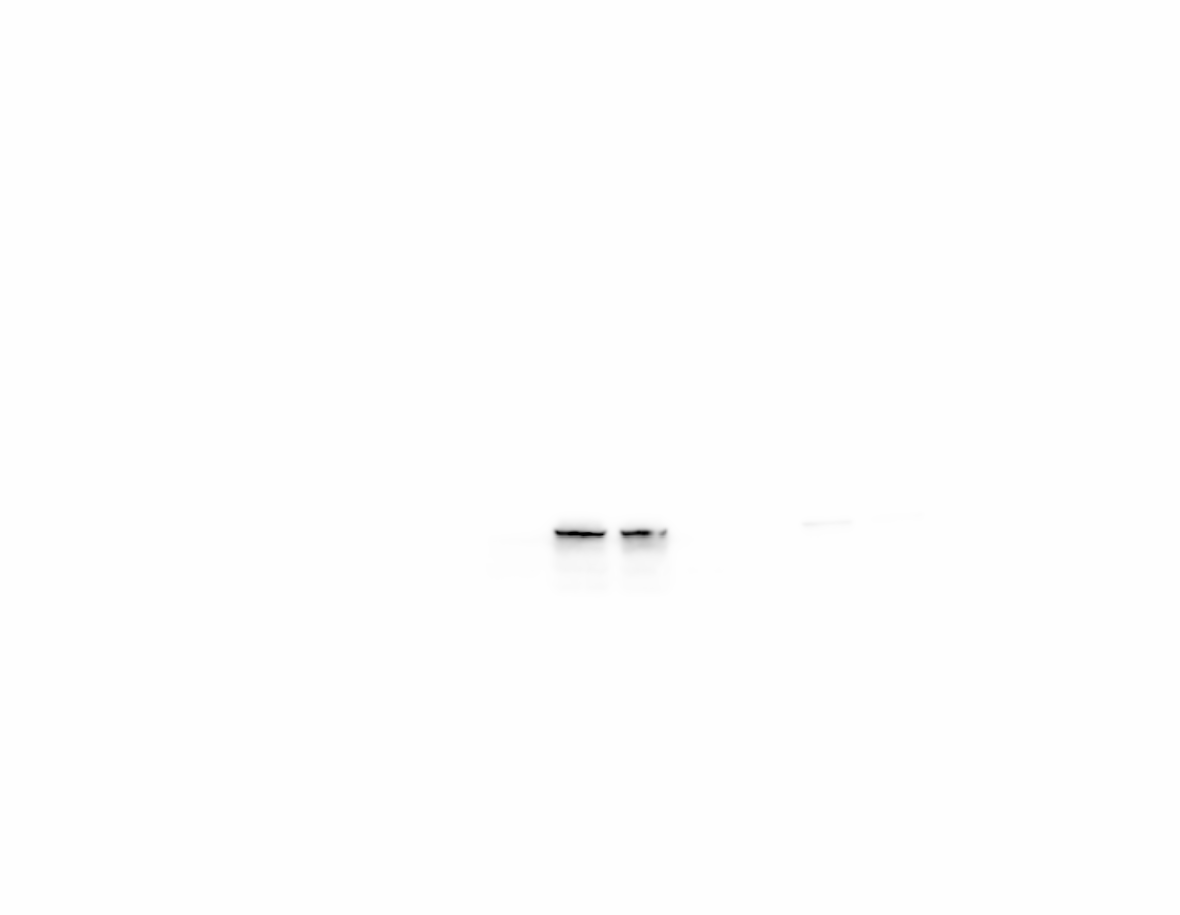

Supplement: Figure 5—source data 1. [file elife-97051-fig5-data1.zip › Figure5B-source data 1. Raw unedited gels for Figure 5B/FLAG_Input_low exp.tif]

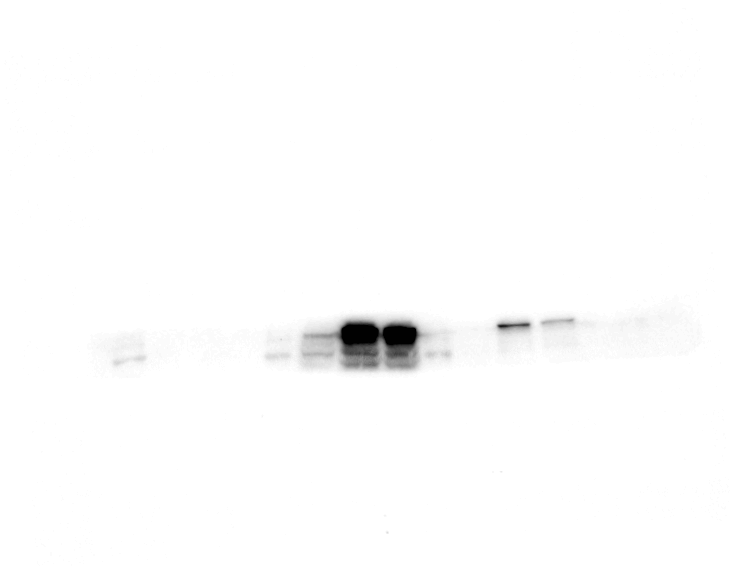

Supplement: Figure 5—source data 1. [file elife-97051-fig5-data1.zip › Figure5B-source data 1. Raw unedited gels for Figure 5B/FLAG_IP_high exp.tif]

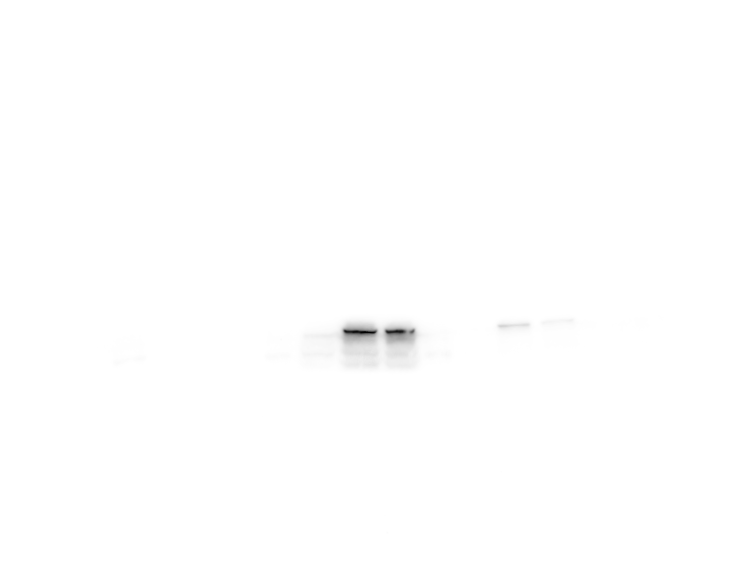

Supplement: Figure 5—source data 1. [file elife-97051-fig5-data1.zip › Figure5B-source data 1. Raw unedited gels for Figure 5B/FLAG_whole.tif]

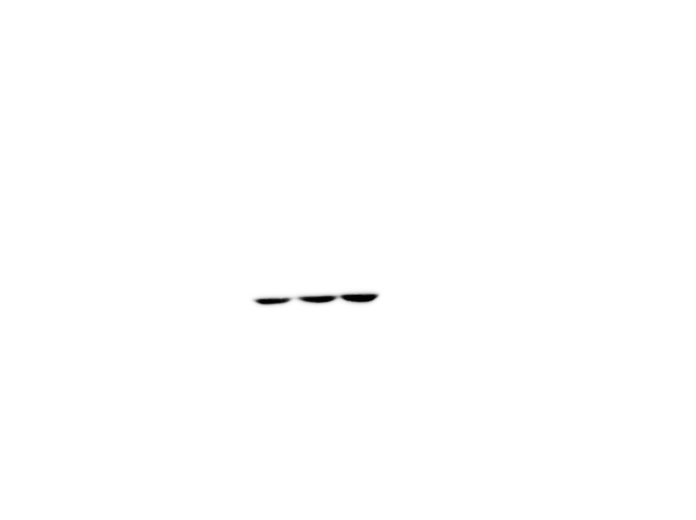

Supplement: Figure 5—source data 1. [file elife-97051-fig5-data1.zip › Figure5B-source data 1. Raw unedited gels for Figure 5B/GAPDH_input.tif]

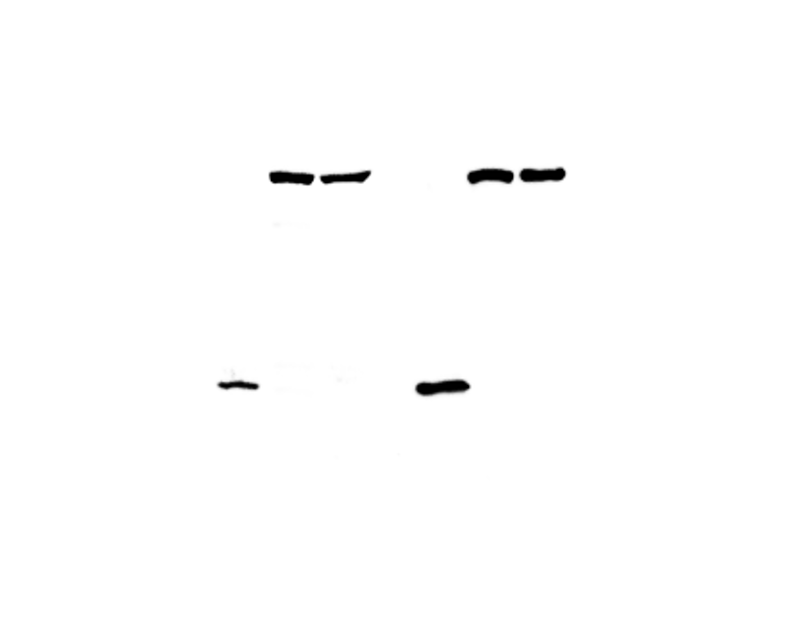

Supplement: Figure 5—source data 1. [file elife-97051-fig5-data1.zip › Figure5B-source data 1. Raw unedited gels for Figure 5B/GST_whole.tif]

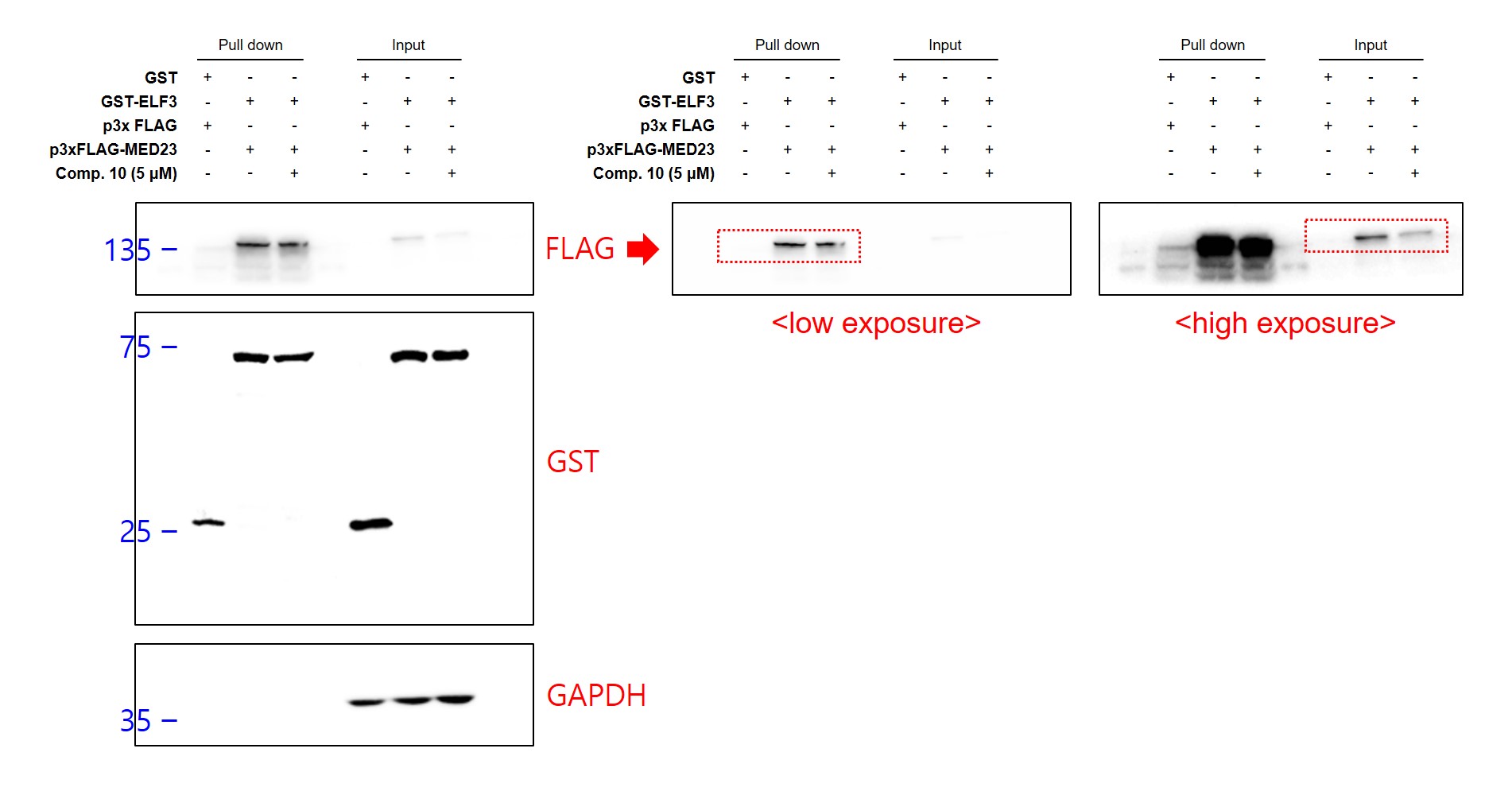

Supplement: Figure 5—source data 2. [file elife-97051-fig5-data2.zip › Figure5B.jpg]

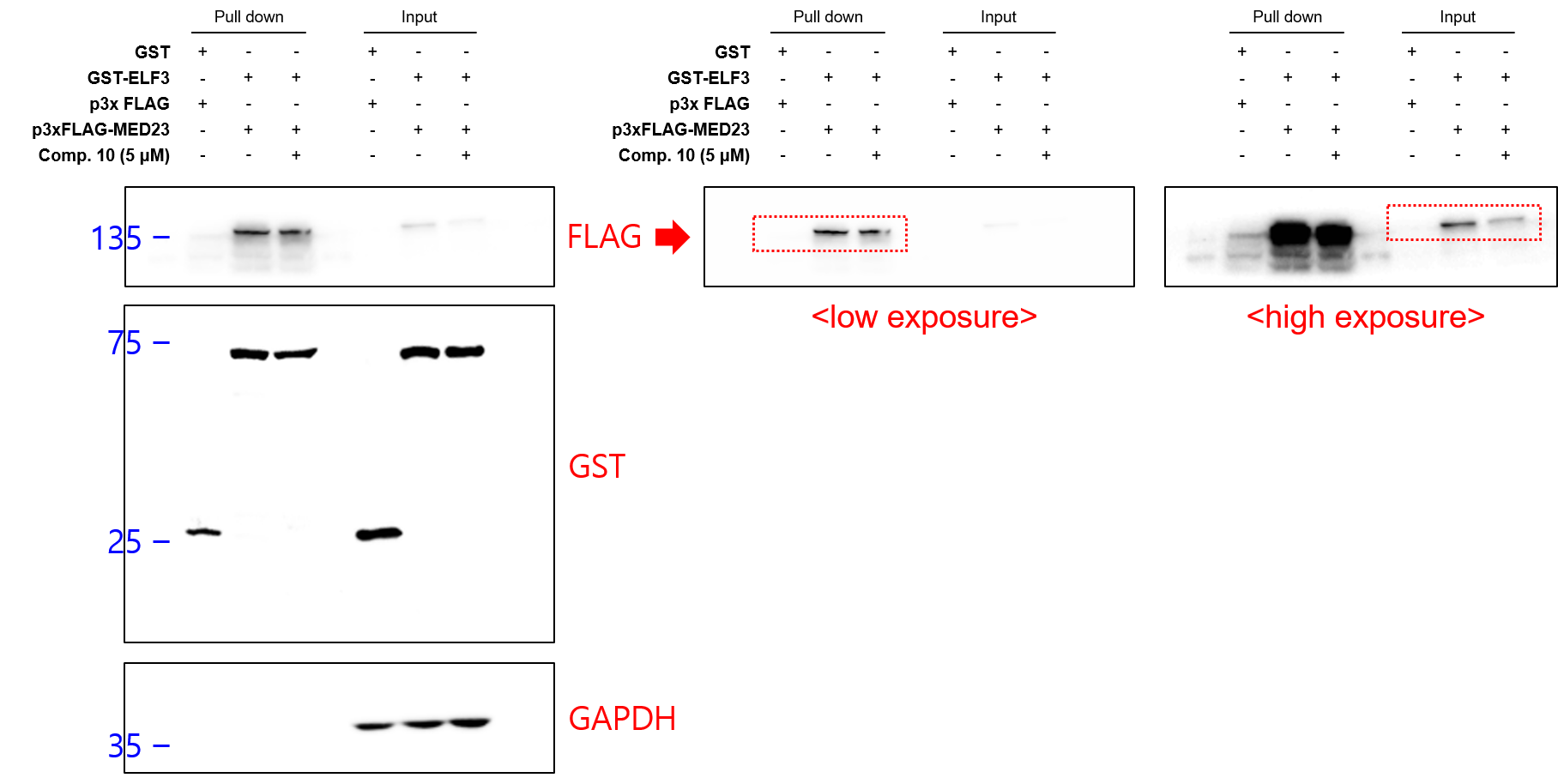

Supplement: Figure 5—source data 2. [file elife-97051-fig5-data2.zip › Figure5B.tif]

Source Data 2

Uncropped blot images of Figure 5C

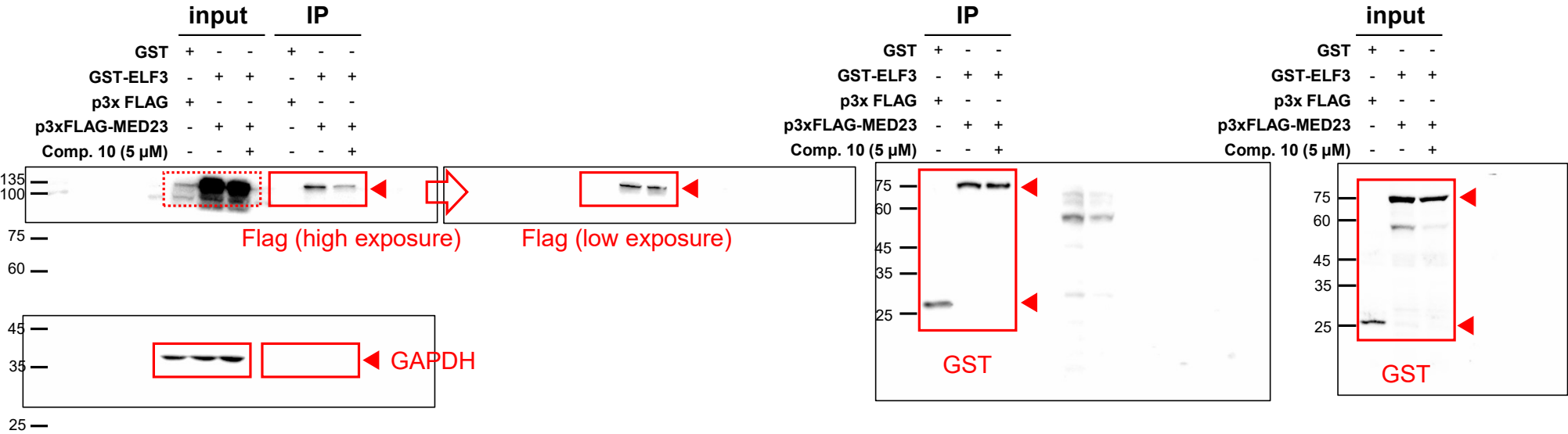

Supplement: Figure 5—source data 2. [file elife-97051-fig5-data2.zip › Source data with molecular weight marker_Part3.pdf]

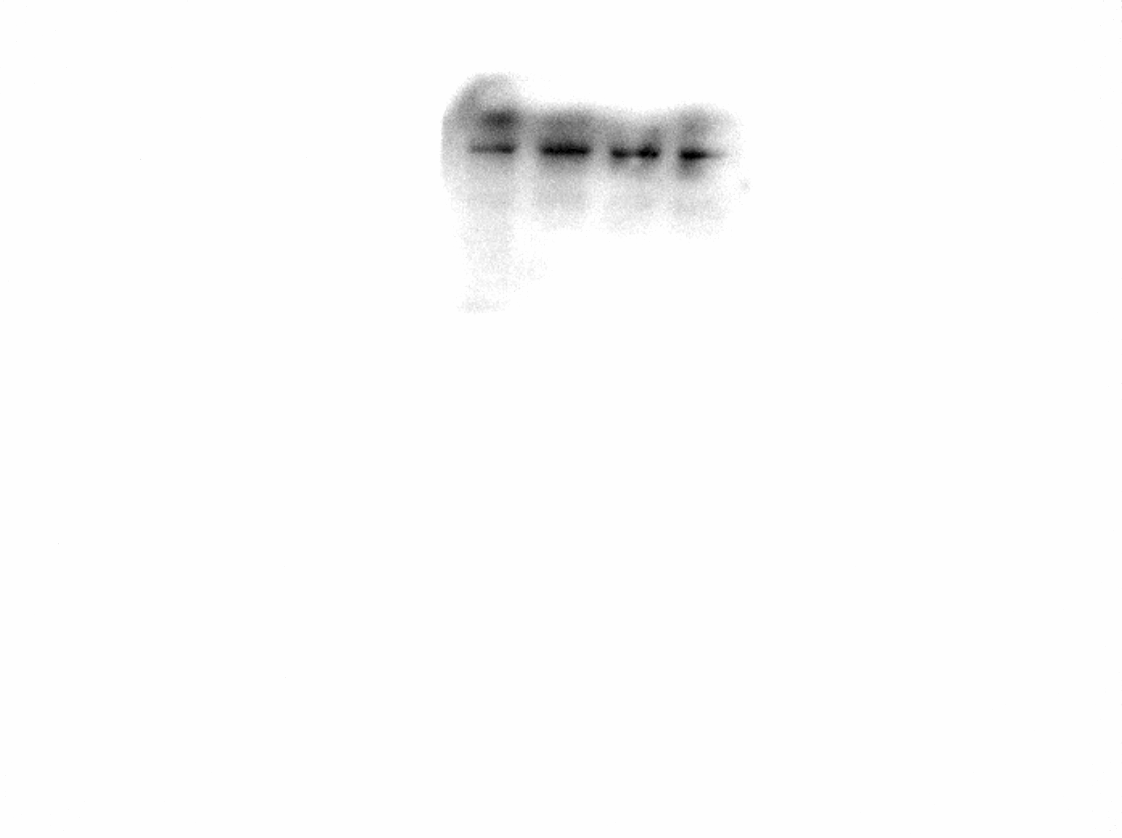

Supplement: Figure 5—source data 3. [file elife-97051-fig5-data3.zip › Figure5F-source data 1. Raw unedited gels for Figure 5F/Figure5F_lower/GAPDH.tif]

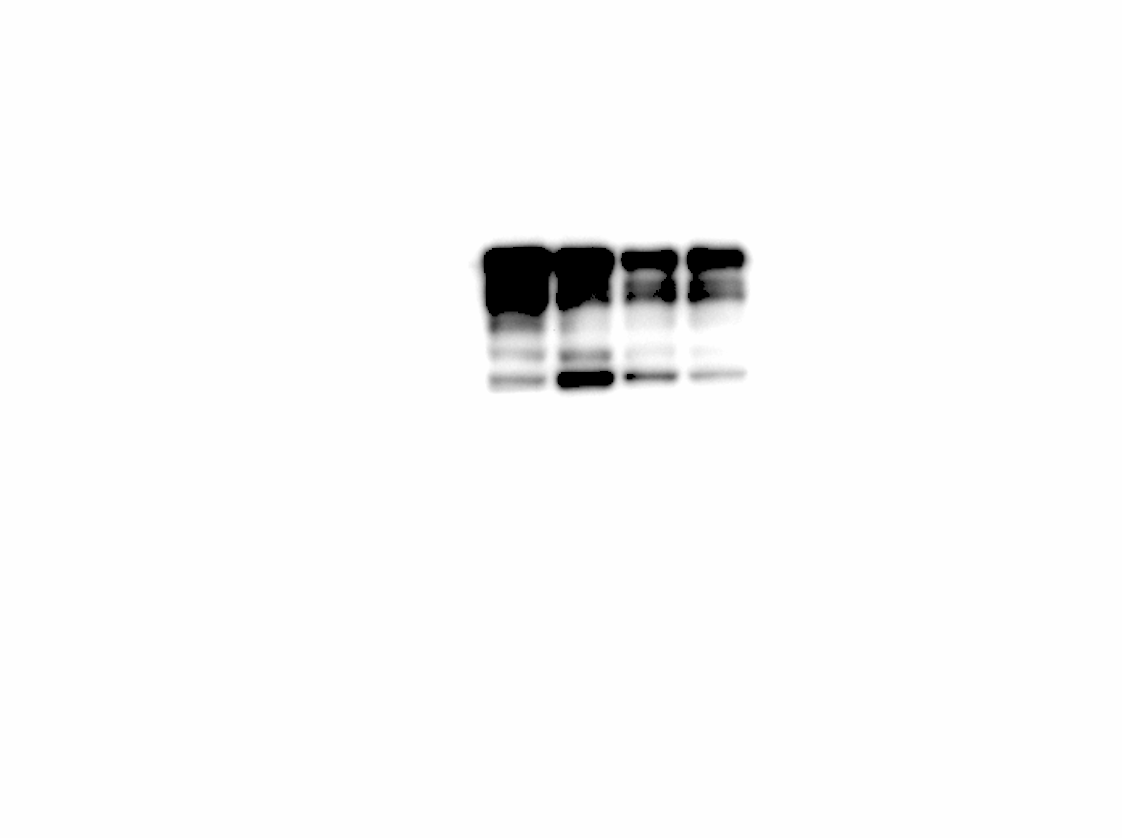

Supplement: Figure 5—source data 3. [file elife-97051-fig5-data3.zip › Figure5F-source data 1. Raw unedited gels for Figure 5F/Figure5F_lower/HER2.tif]

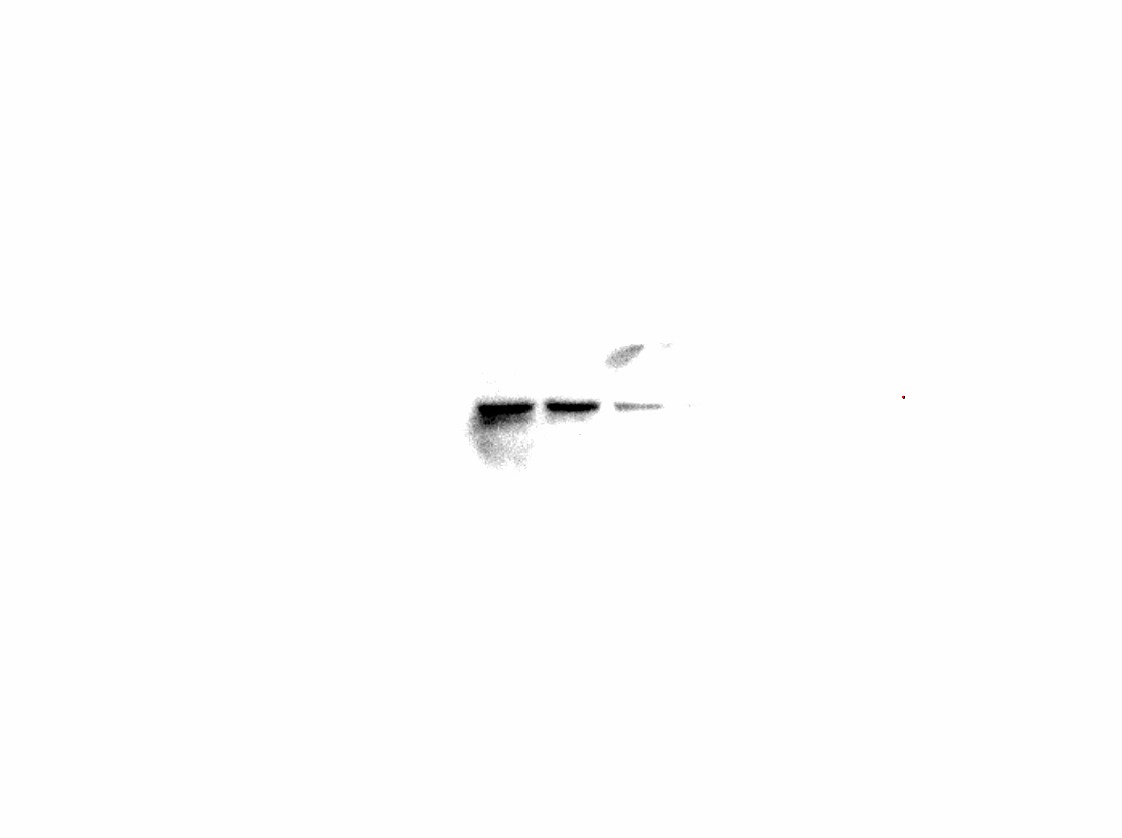

Supplement: Figure 5—source data 3. [file elife-97051-fig5-data3.zip › Figure5F-source data 1. Raw unedited gels for Figure 5F/Figure5F_lower/pAKT.tif]

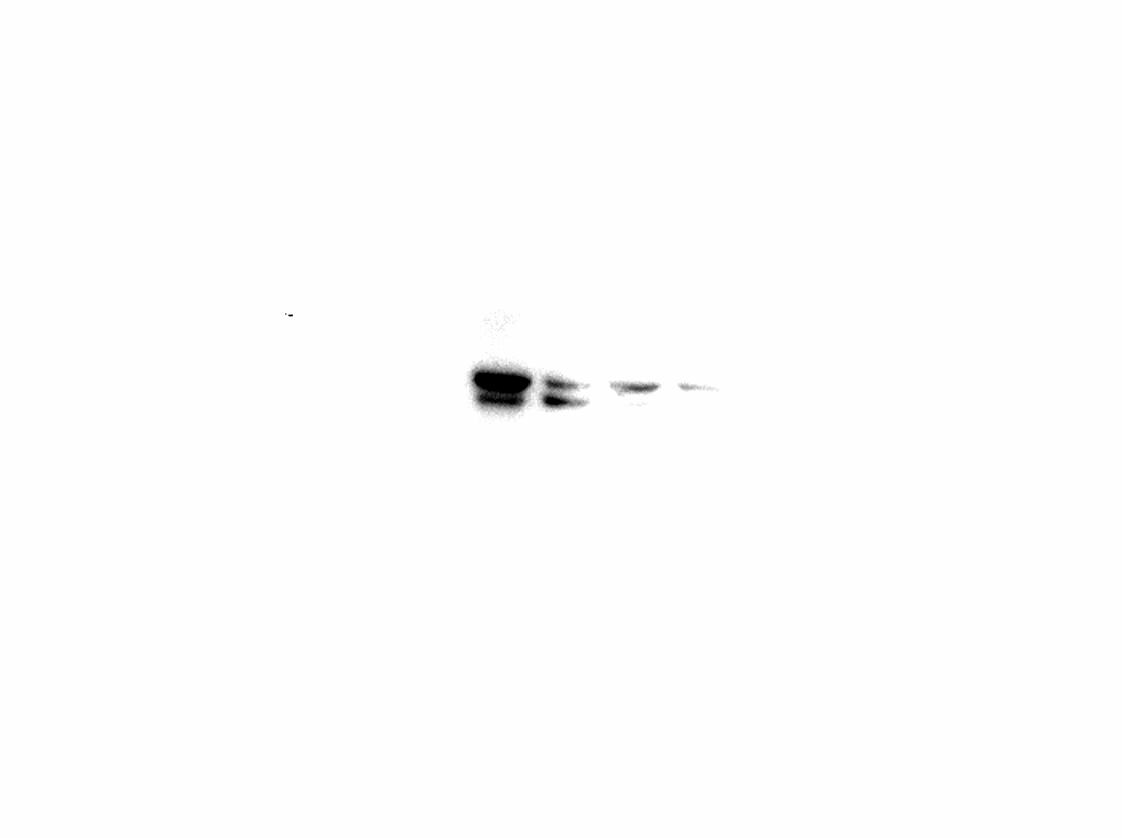

Supplement: Figure 5—source data 3. [file elife-97051-fig5-data3.zip › Figure5F-source data 1. Raw unedited gels for Figure 5F/Figure5F_lower/pMAPK.tif]

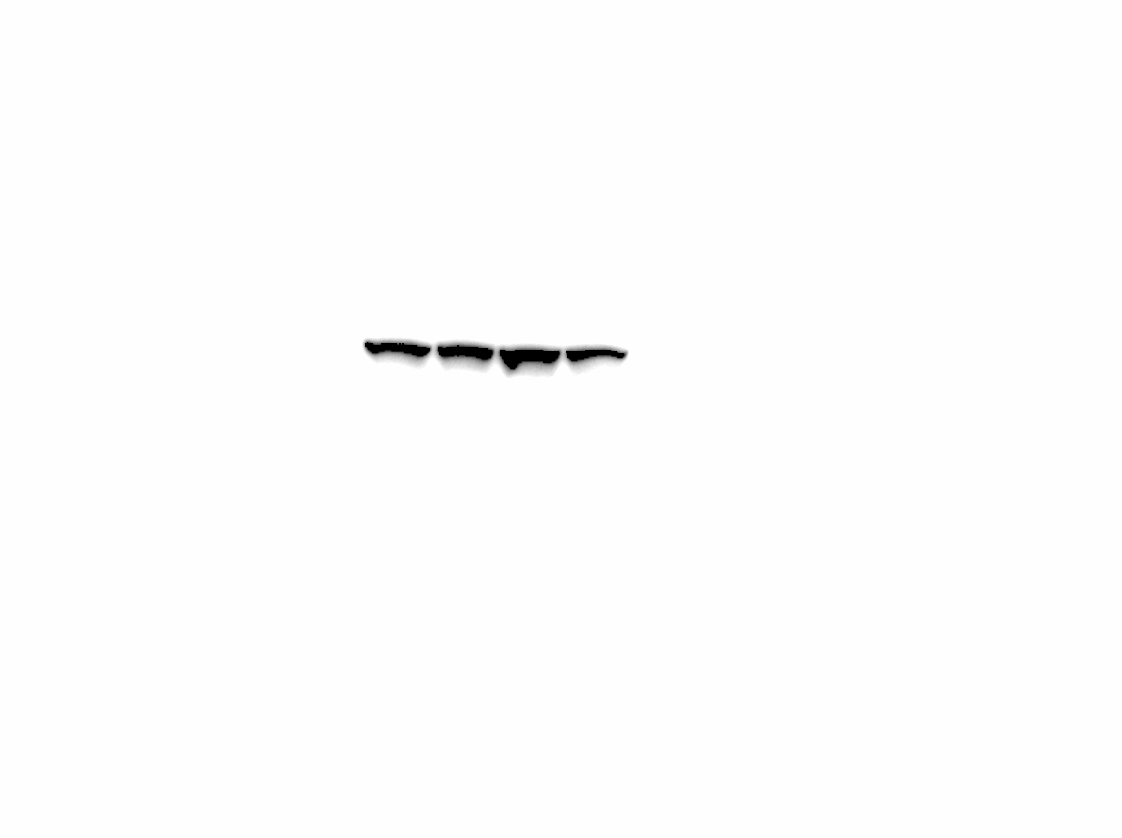

Supplement: Figure 5—source data 3. [file elife-97051-fig5-data3.zip › Figure5F-source data 1. Raw unedited gels for Figure 5F/Figure5F_lower/Vinculin.tif]

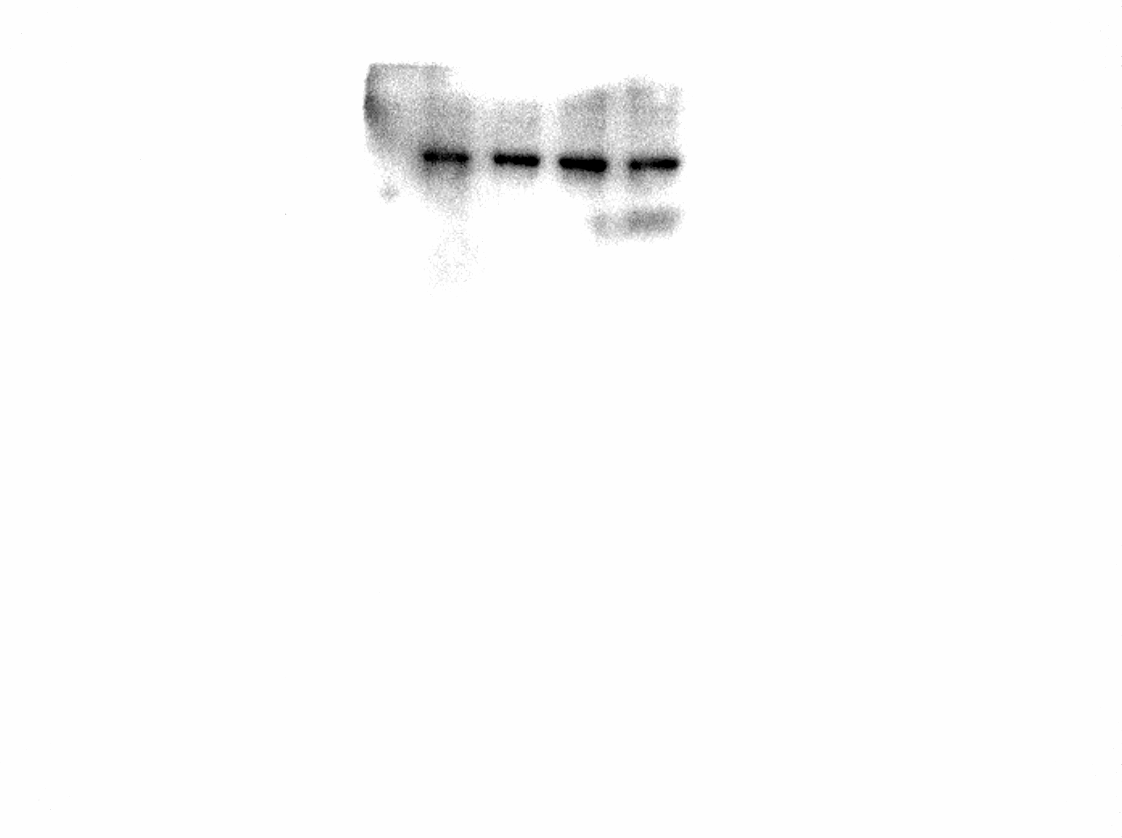

Supplement: Figure 5—source data 3. [file elife-97051-fig5-data3.zip › Figure5F-source data 1. Raw unedited gels for Figure 5F/Figure5F_upper/GAPDH(II).tif]

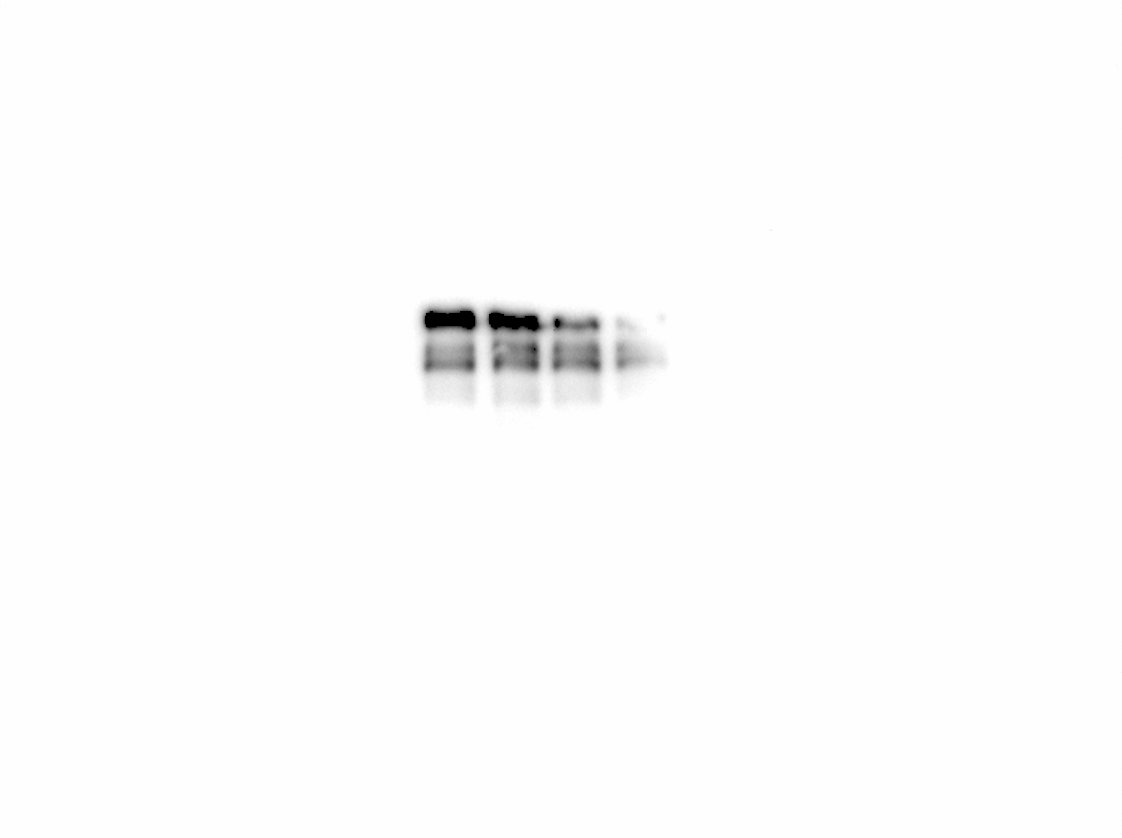

Supplement: Figure 5—source data 3. [file elife-97051-fig5-data3.zip › Figure5F-source data 1. Raw unedited gels for Figure 5F/Figure5F_upper/HER2.tif]

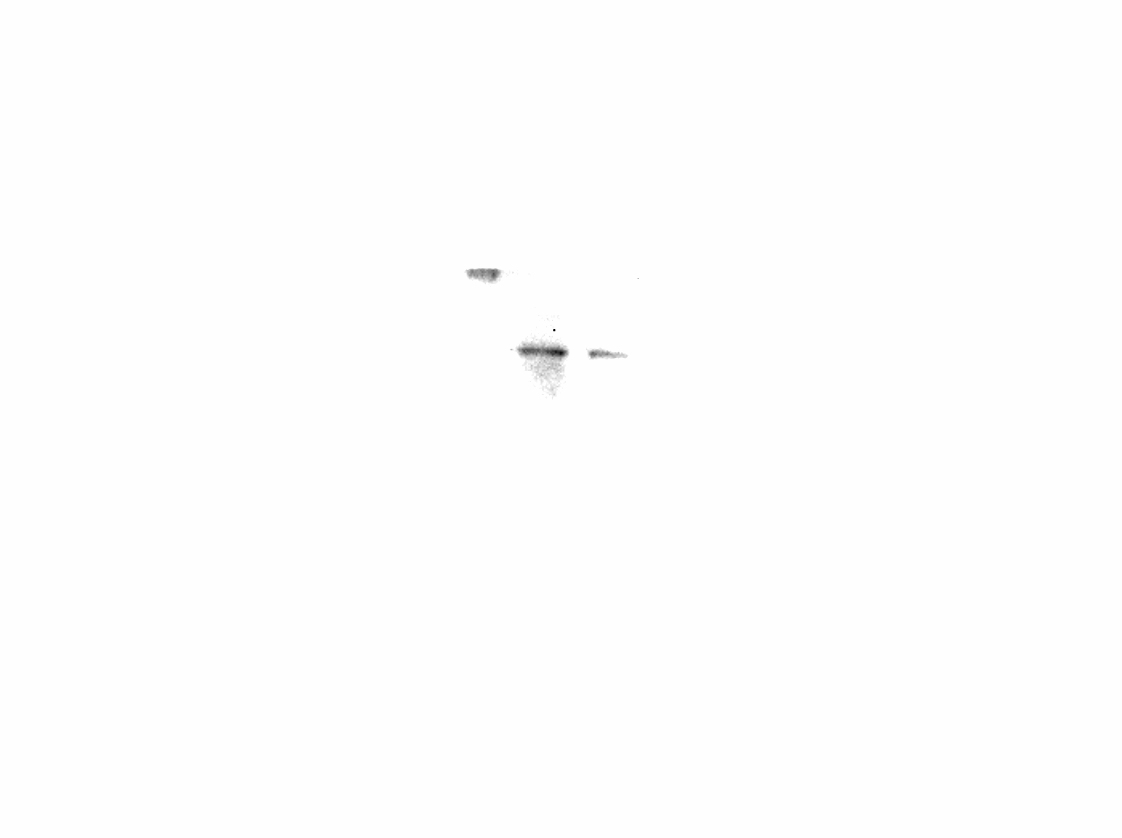

Supplement: Figure 5—source data 3. [file elife-97051-fig5-data3.zip › Figure5F-source data 1. Raw unedited gels for Figure 5F/Figure5F_upper/pAKT.tif]

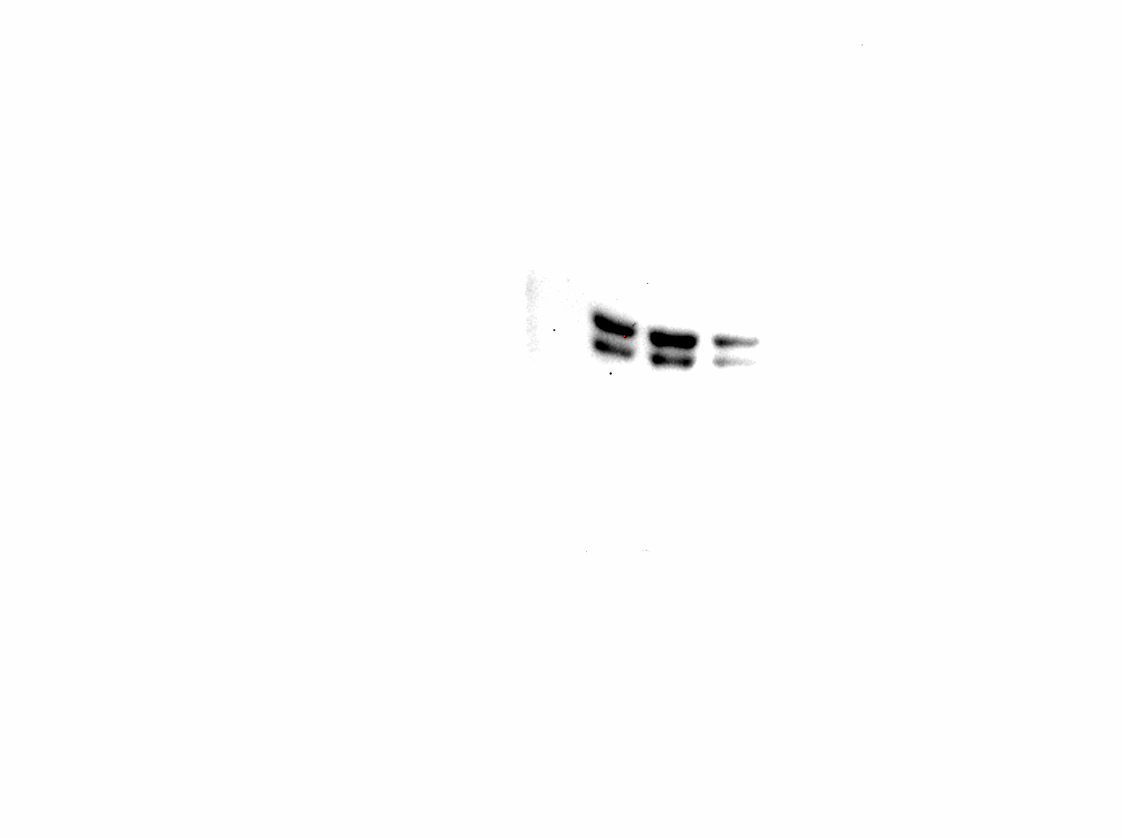

Supplement: Figure 5—source data 3. [file elife-97051-fig5-data3.zip › Figure5F-source data 1. Raw unedited gels for Figure 5F/Figure5F_upper/pMAPK.tif]

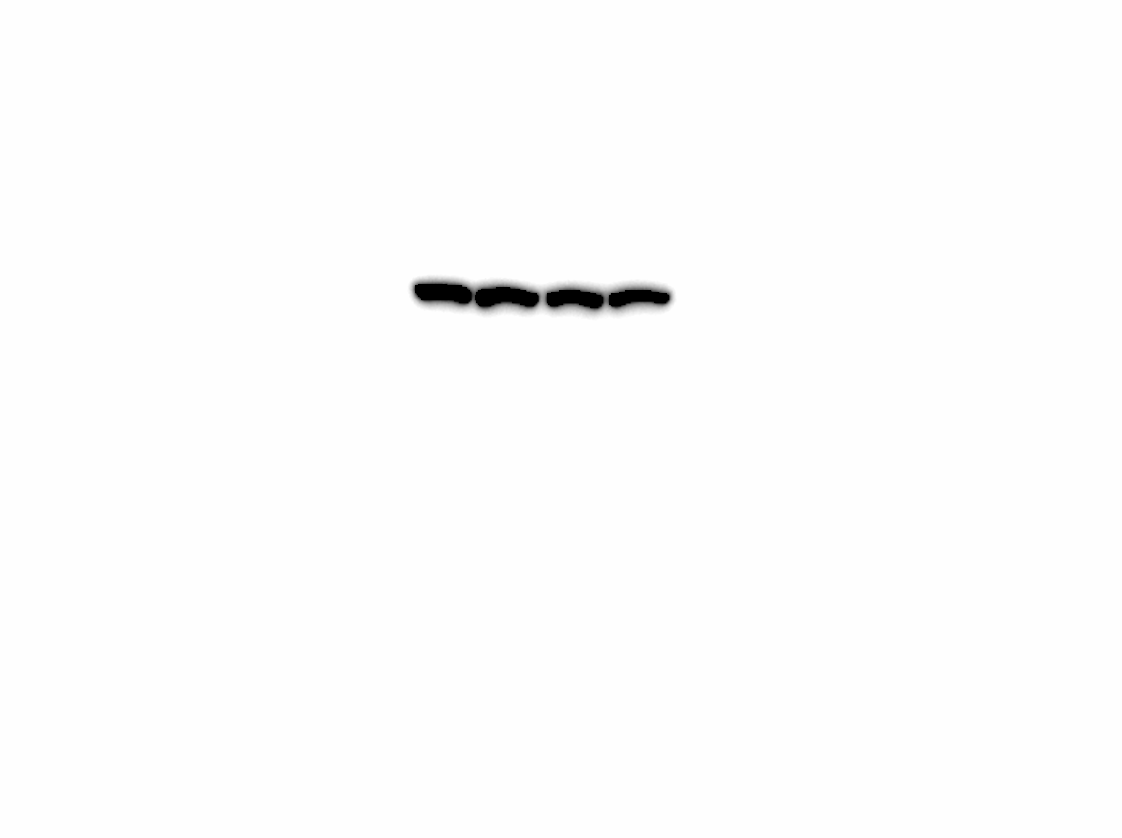

Supplement: Figure 5—source data 3. [file elife-97051-fig5-data3.zip › Figure5F-source data 1. Raw unedited gels for Figure 5F/Figure5F_upper/vinculin.tif]

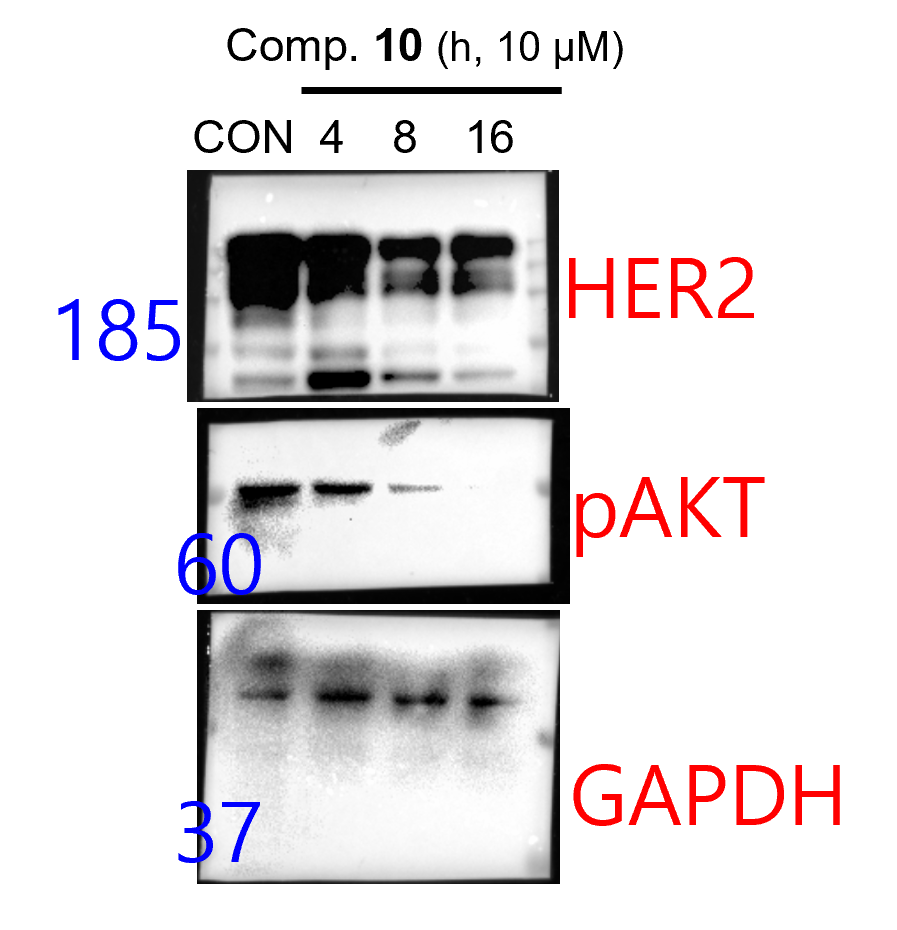

Supplement: Figure 5—source data 4. [file elife-97051-fig5-data4.zip › lower_1.tif]

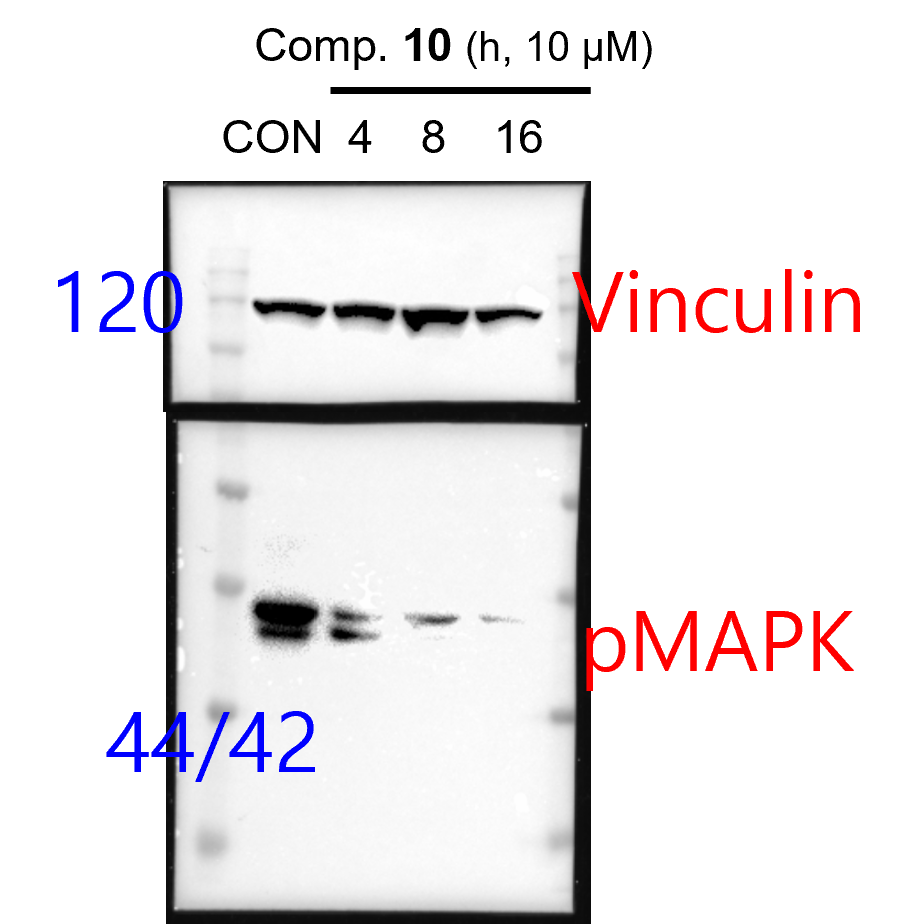

Supplement: Figure 5—source data 4. [file elife-97051-fig5-data4.zip › lower_2.tif]

## Source Data 2

### Uncropped blot images of Figure 5F

*upper*

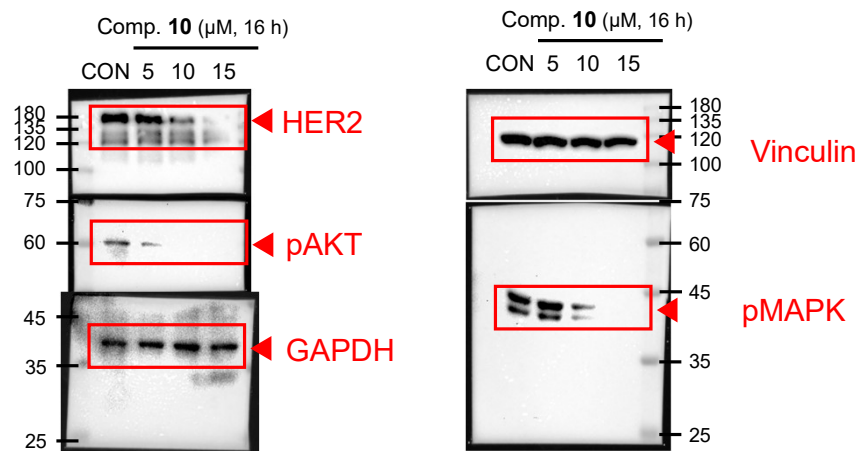

*lower*

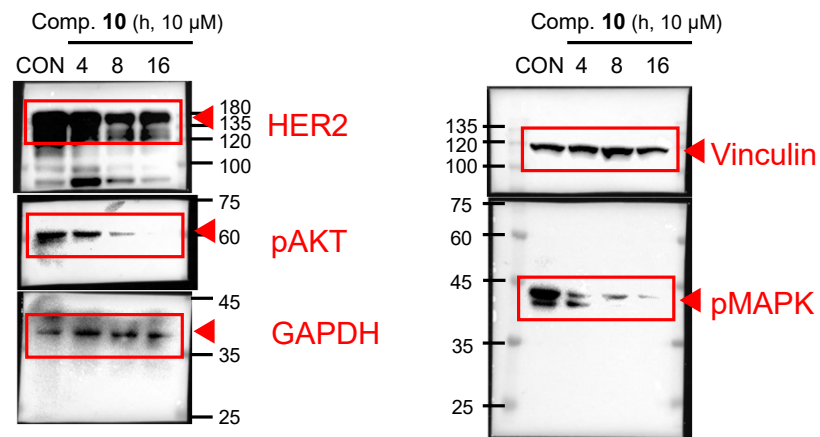

Supplement: Figure 5—source data 4. [file elife-97051-fig5-data4.zip › Source data with molecular weight marker_Part4.pdf]

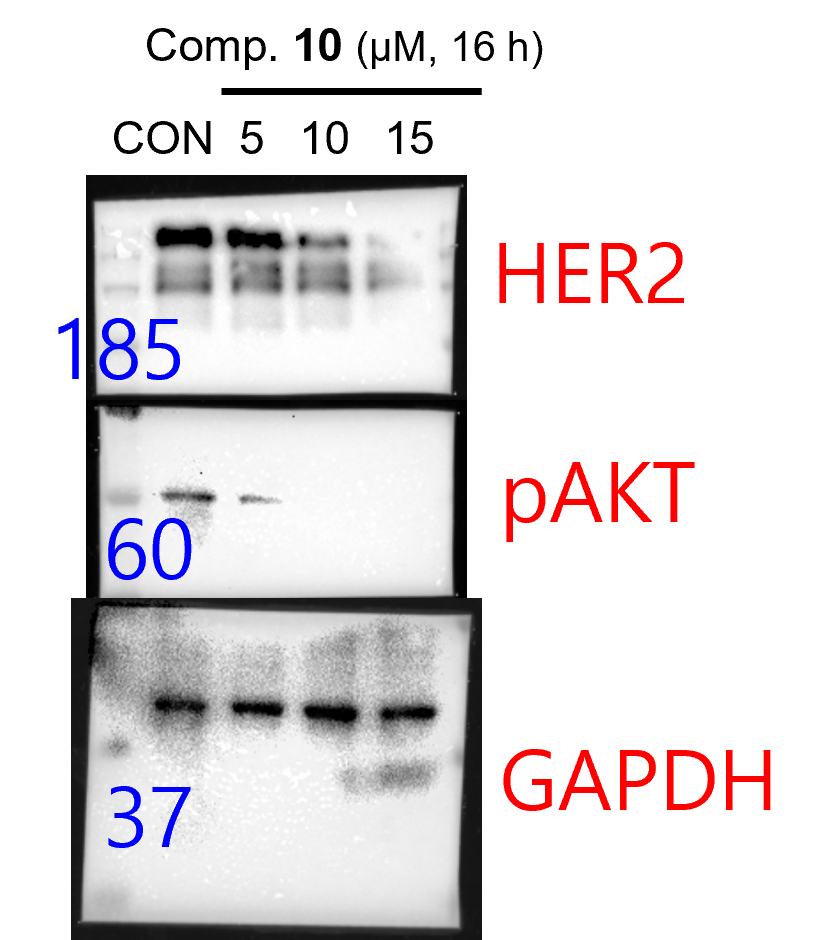

Supplement: Figure 5—source data 4. [file elife-97051-fig5-data4.zip › upper_1.tif]

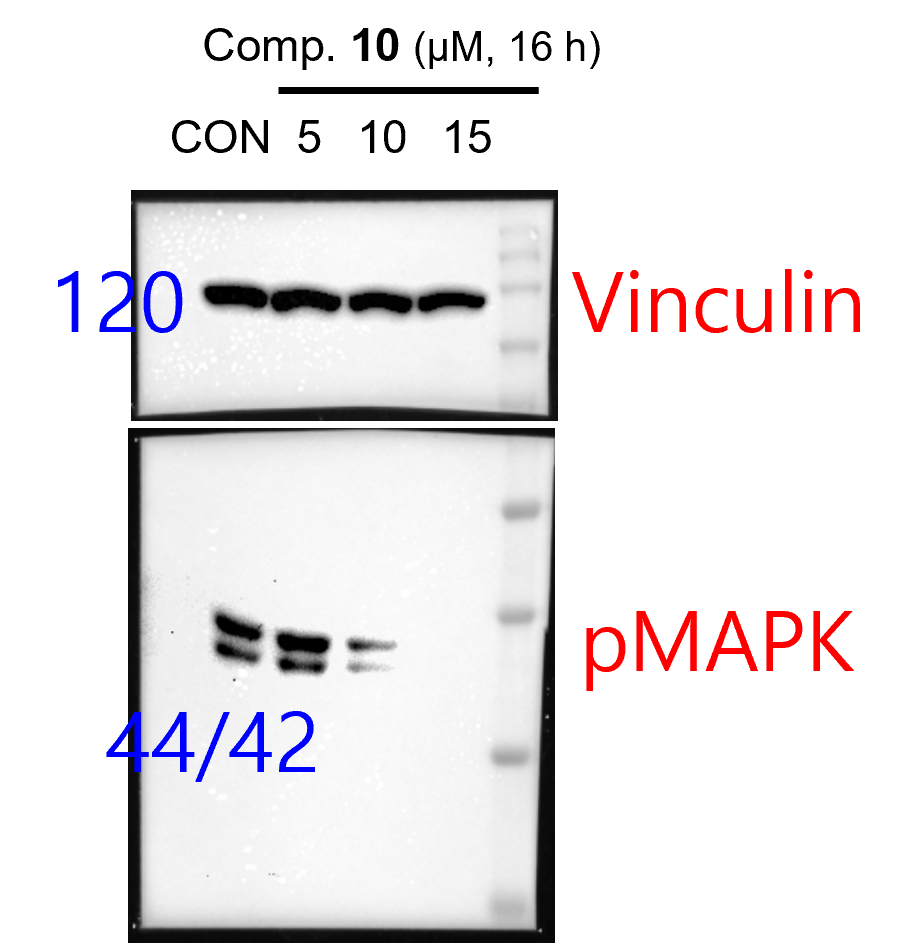

Supplement: Figure 5—source data 4. [file elife-97051-fig5-data4.zip › upper_2.tif]

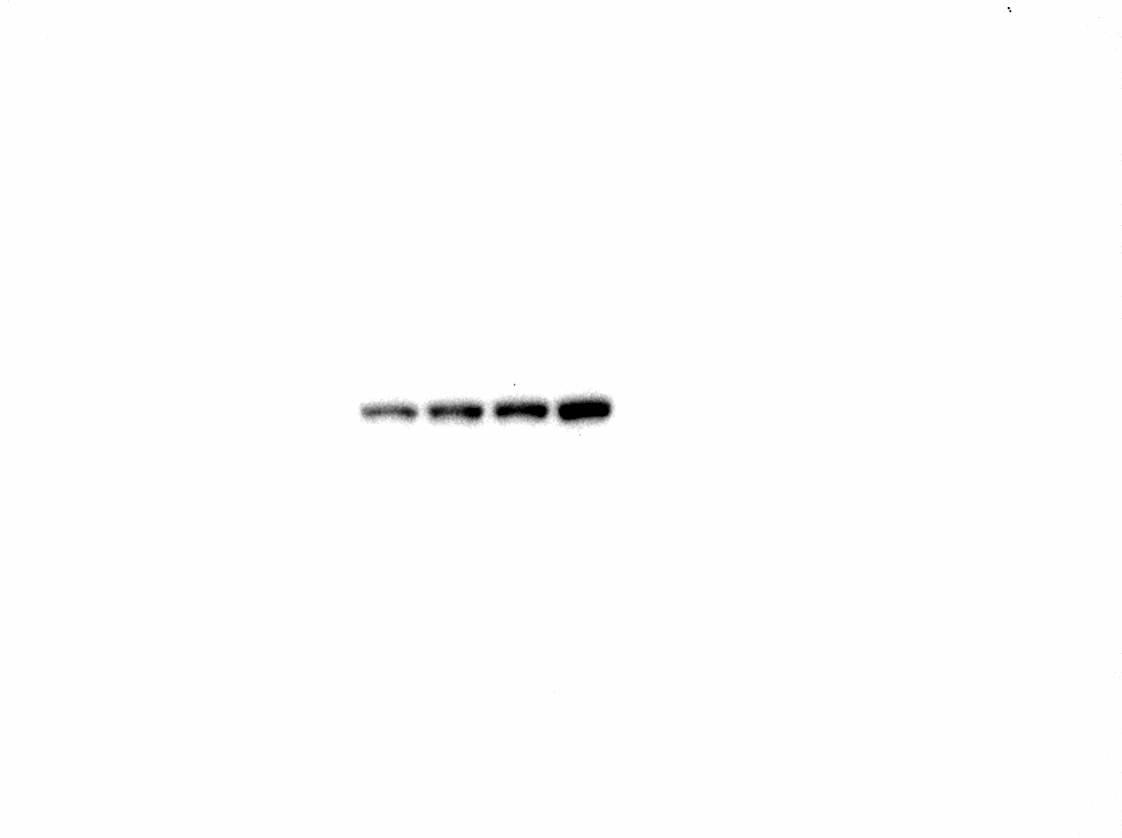

Supplement: Figure 6—source data 1. [file elife-97051-fig6-data1.zip › Figure6B-source data 1. Raw unedited gels for Figure 6B/lower_cPARP.tif]

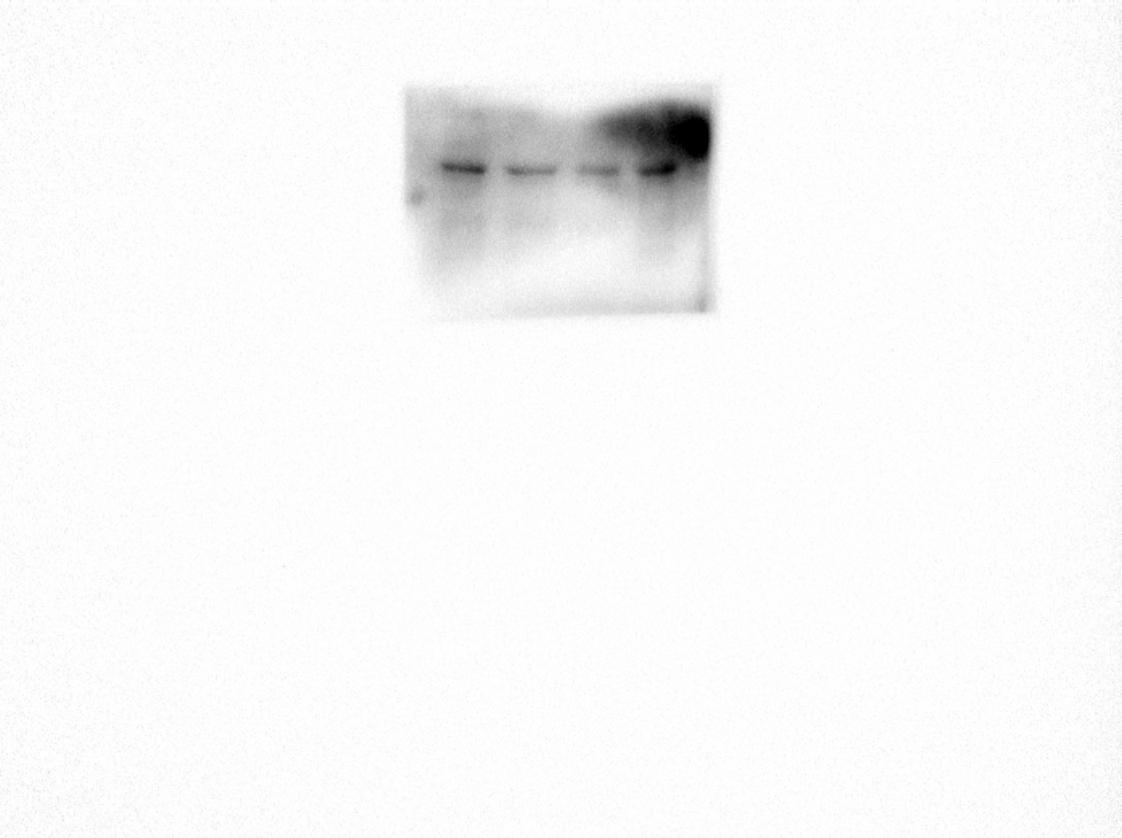

Supplement: Figure 6—source data 1. [file elife-97051-fig6-data1.zip › Figure6B-source data 1. Raw unedited gels for Figure 6B/lower_GAPDH.tif]

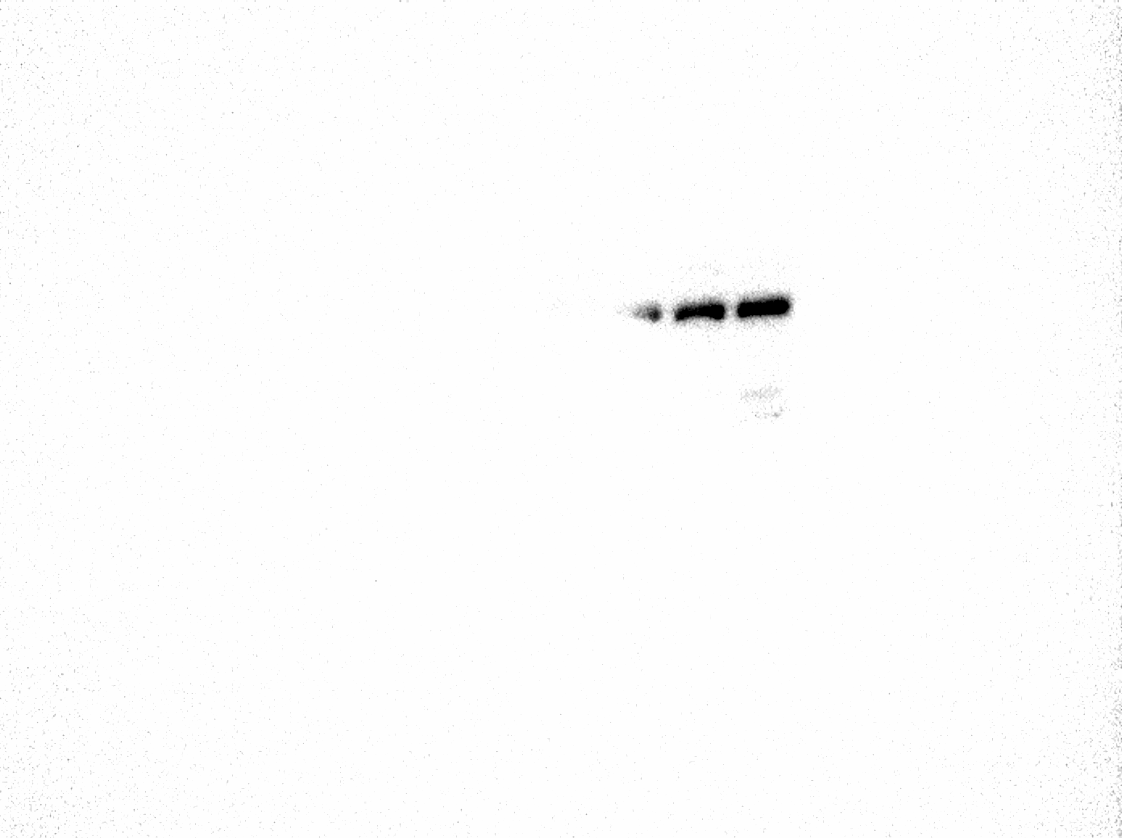

Supplement: Figure 6—source data 1. [file elife-97051-fig6-data1.zip › Figure6B-source data 1. Raw unedited gels for Figure 6B/upper_cPARP.tif]

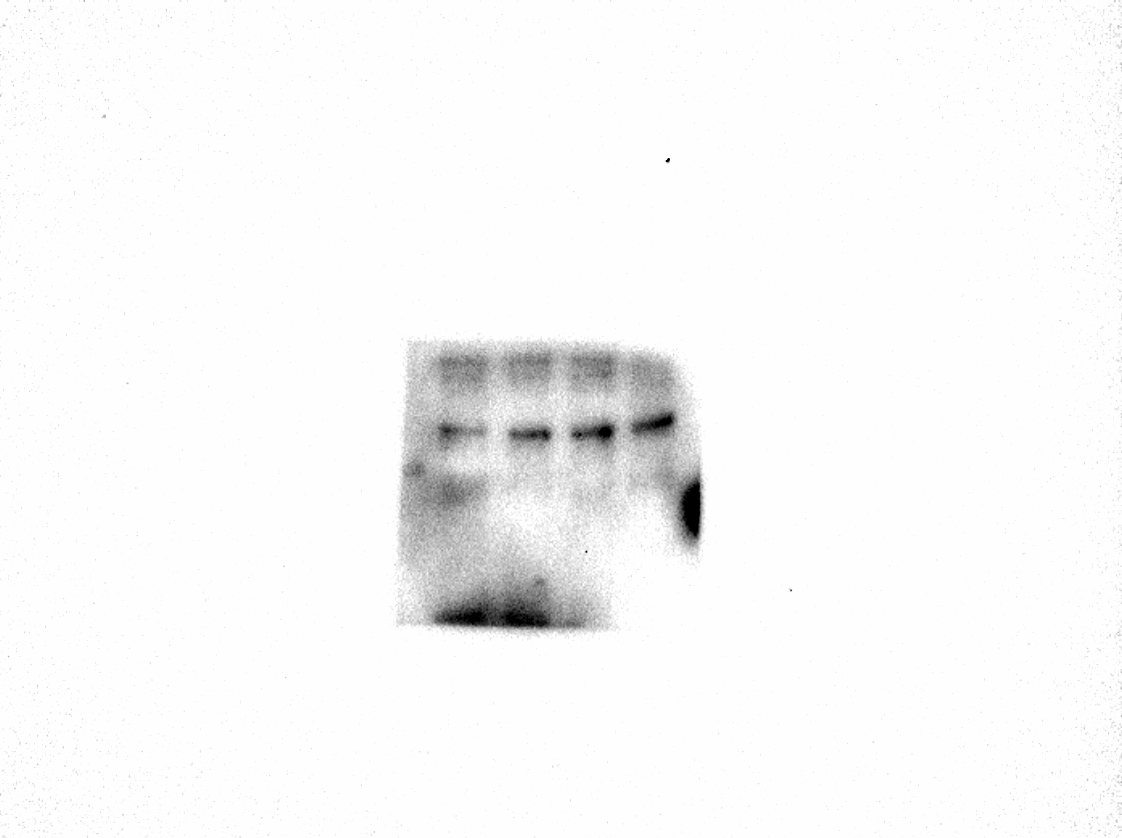

Supplement: Figure 6—source data 1. [file elife-97051-fig6-data1.zip › Figure6B-source data 1. Raw unedited gels for Figure 6B/upper_GAPDH.tif]

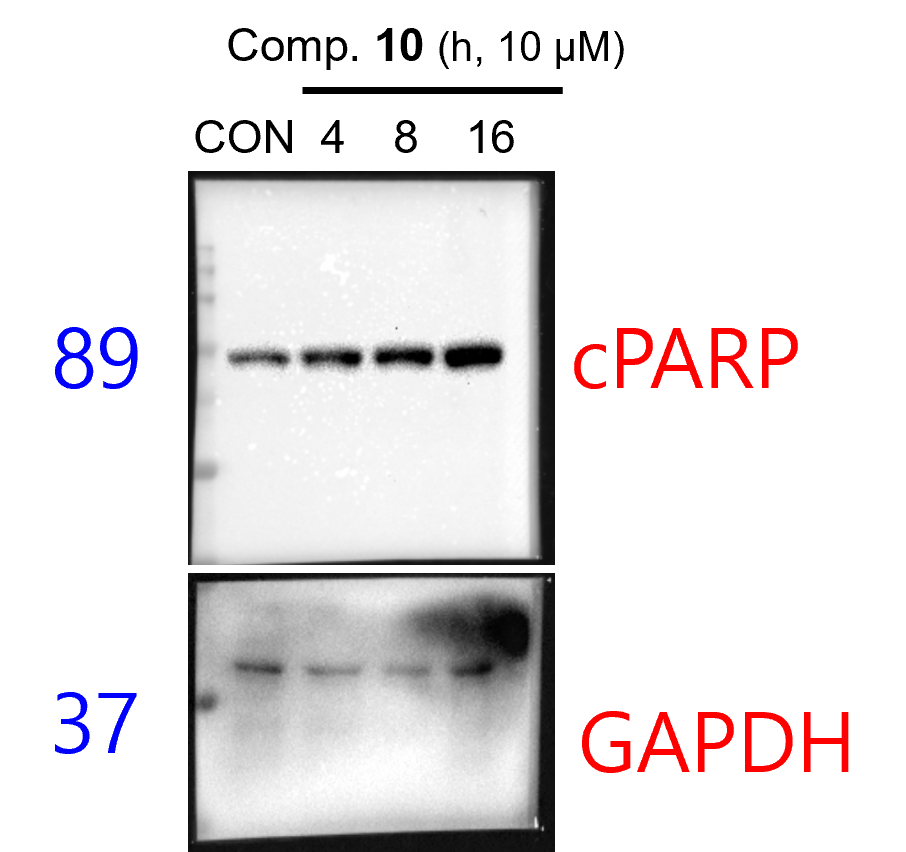

Supplement: Figure 6—source data 2. [file elife-97051-fig6-data2.zip › lower_1.tif]

## Source Data 2

### Uncropped blot images of Figure 6B

*upper*

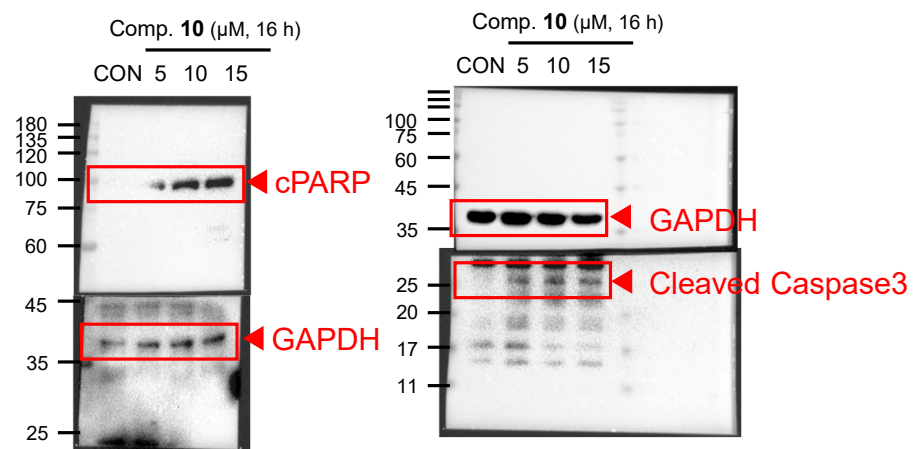

*lower*

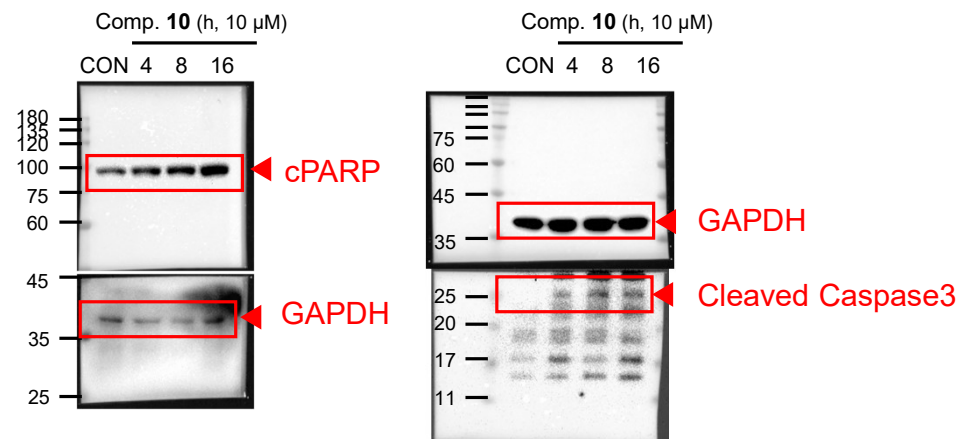

Supplement: Figure 6—source data 2. [file elife-97051-fig6-data2.zip › Source data with molecular weight marker_Part5.pdf]

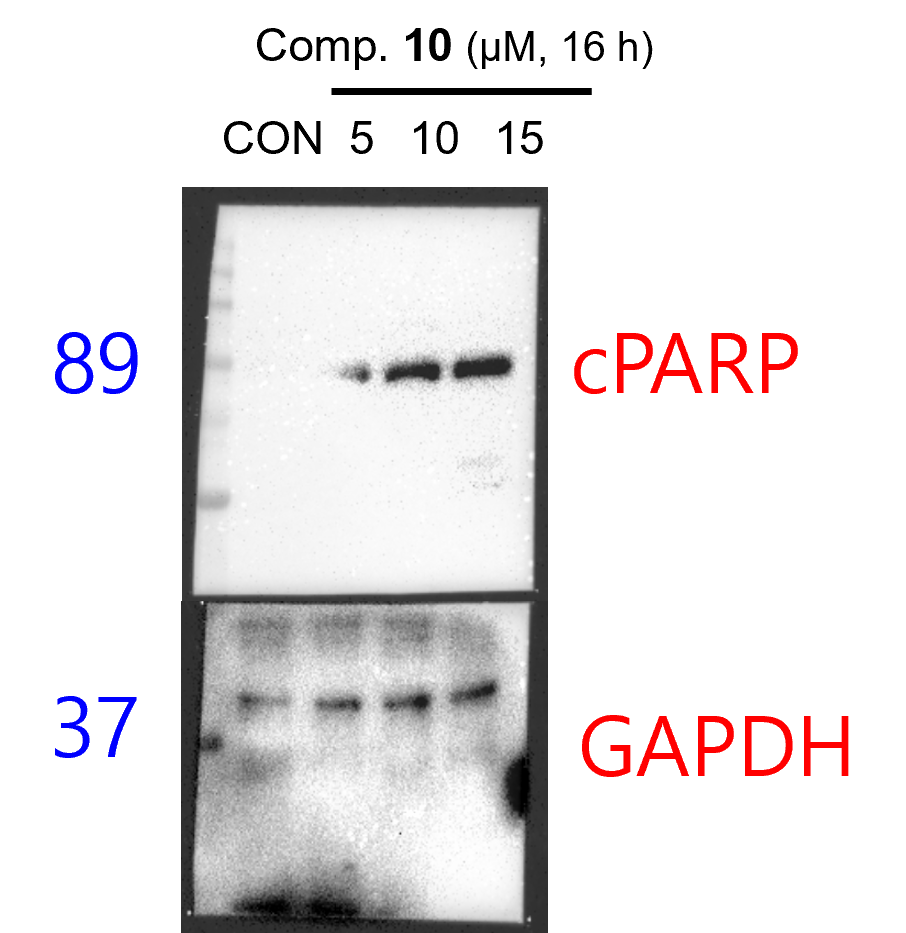

Supplement: Figure 6—source data 2. [file elife-97051-fig6-data2.zip › upper_1.tif]

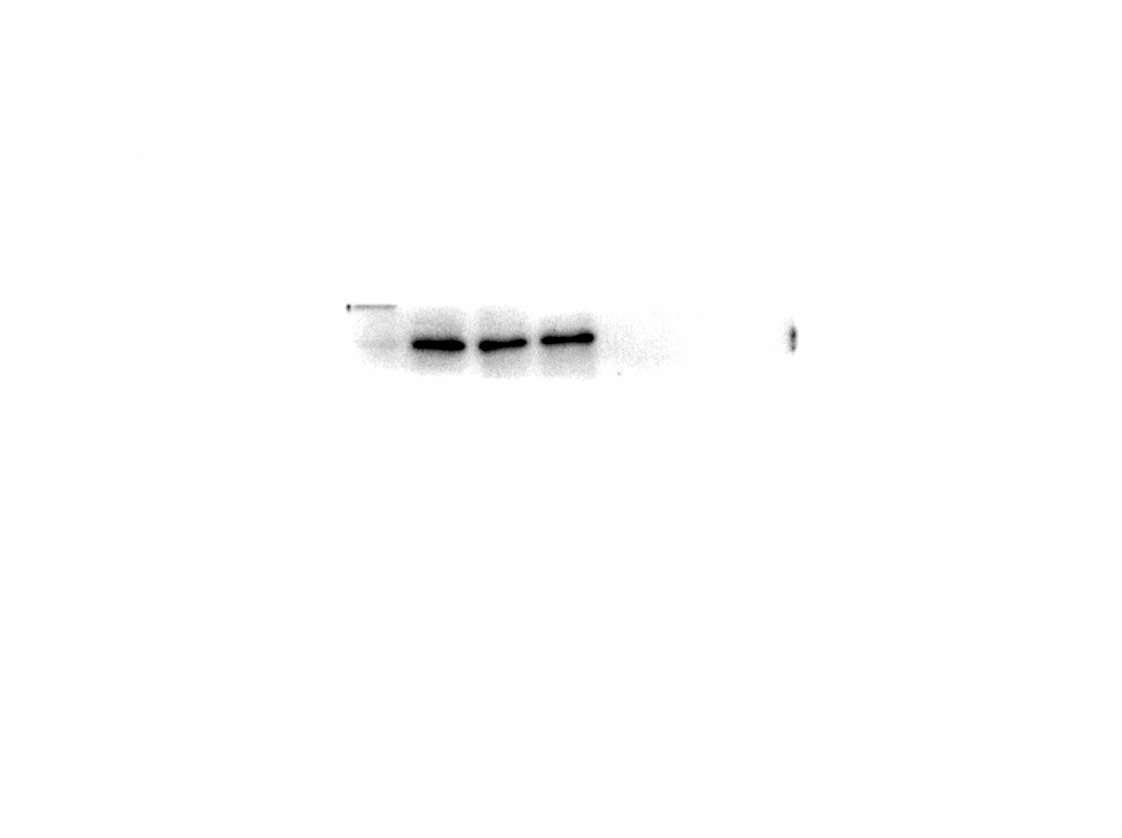

Supplement: Figure 7—source data 1. [file elife-97051-fig7-data1.zip › Figure7B-source data 1. Raw unedited gels for Figure 7B/AKT.tif]

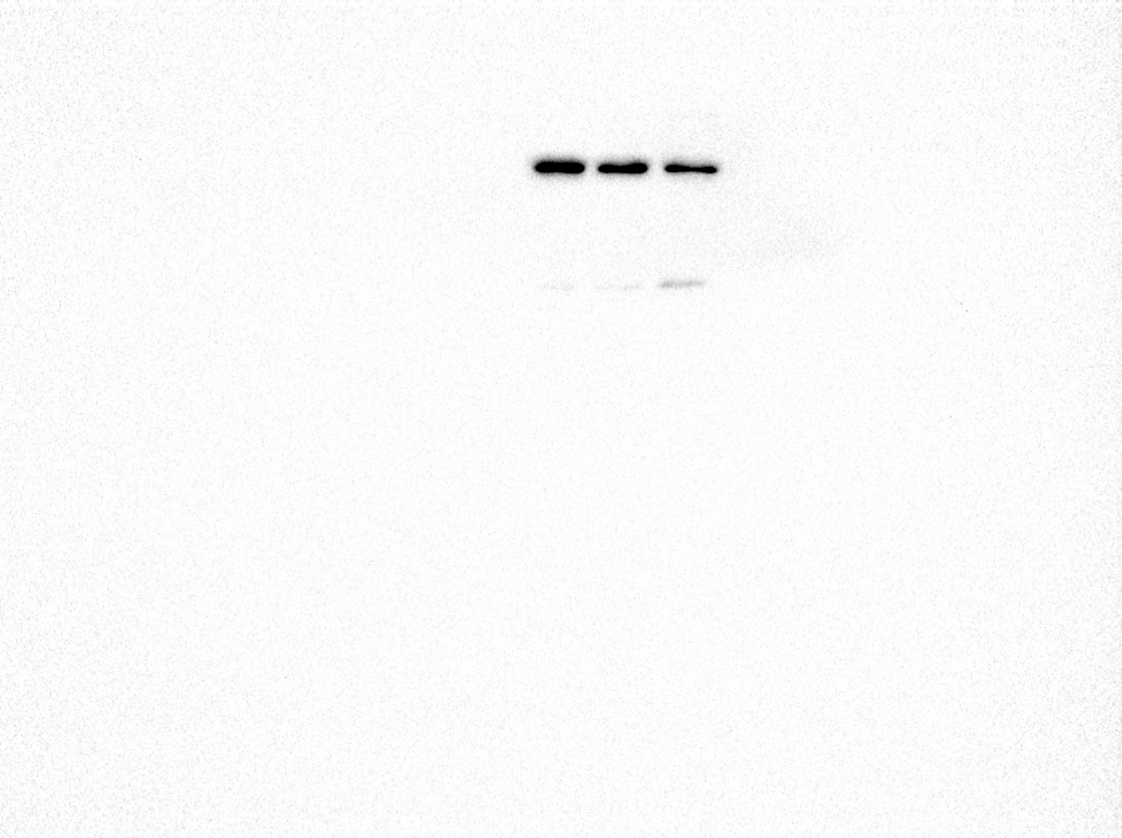

Supplement: Figure 7—source data 1. [file elife-97051-fig7-data1.zip › Figure7B-source data 1. Raw unedited gels for Figure 7B/GAPDH1.tif]

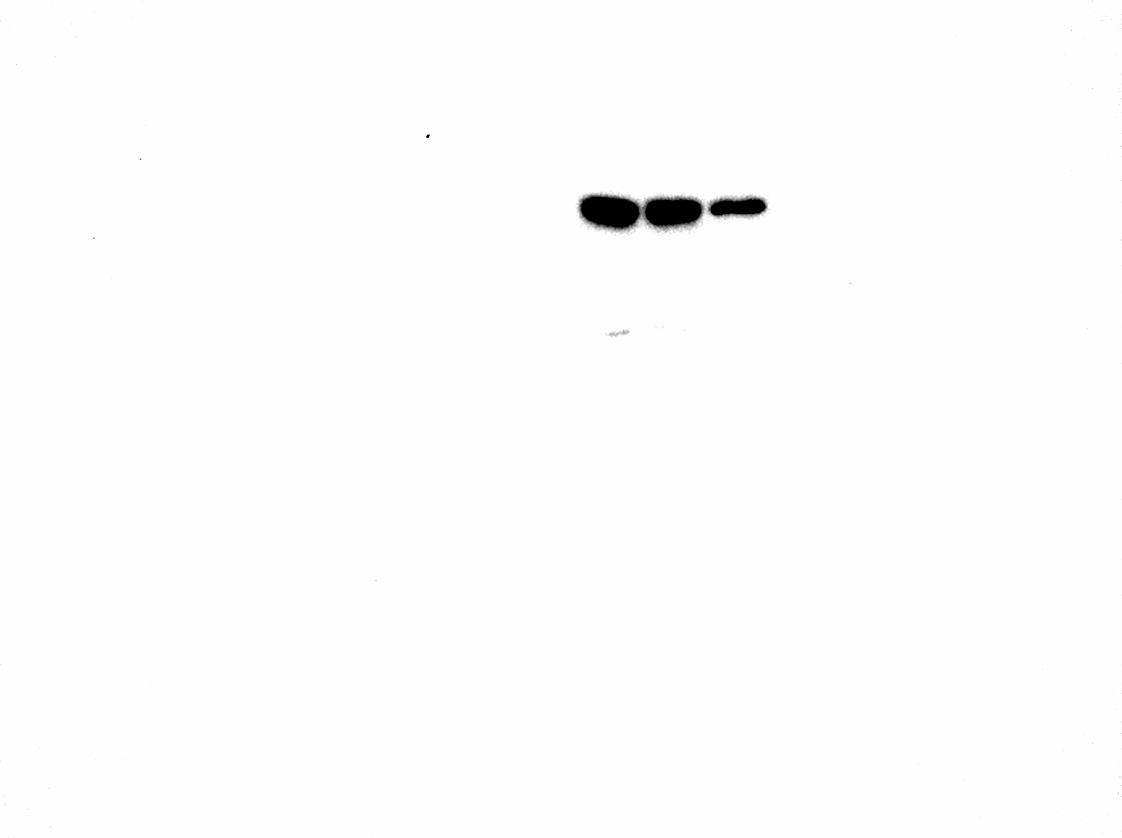

Supplement: Figure 7—source data 1. [file elife-97051-fig7-data1.zip › Figure7B-source data 1. Raw unedited gels for Figure 7B/GAPDH2.tif]

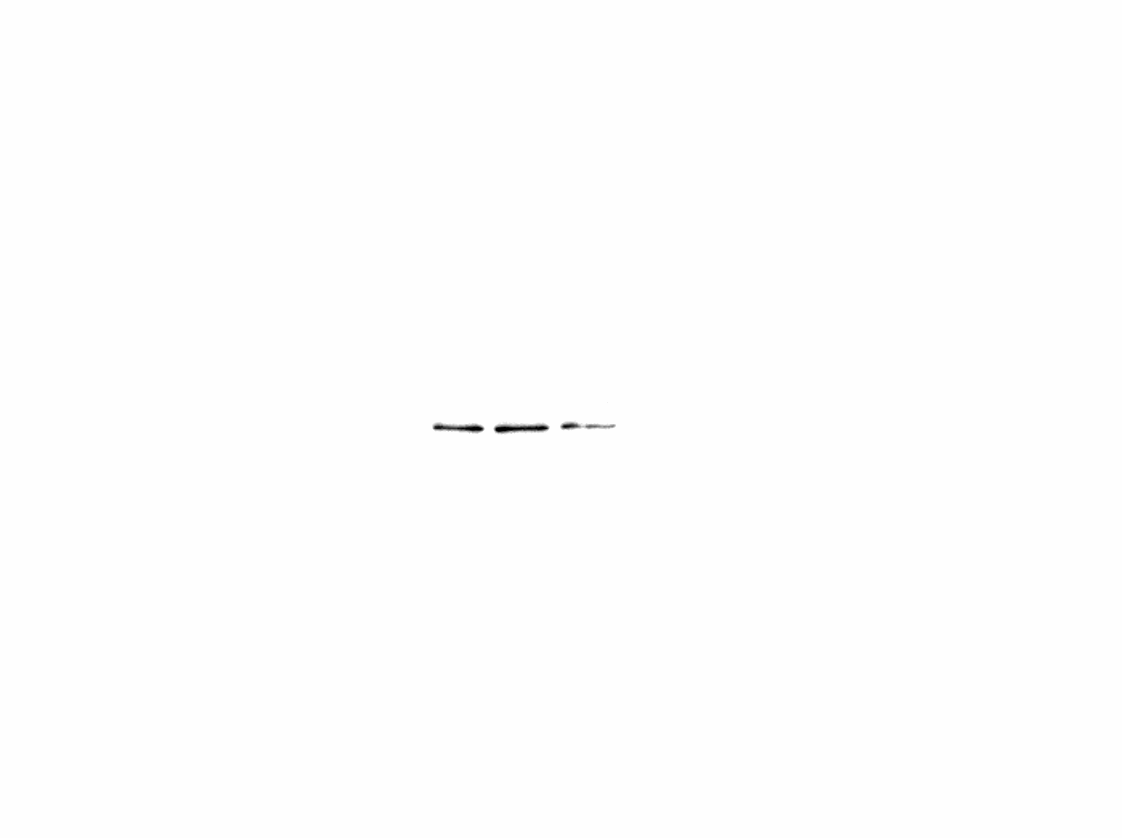

Supplement: Figure 7—source data 1. [file elife-97051-fig7-data1.zip › Figure7B-source data 1. Raw unedited gels for Figure 7B/HER2.tif]

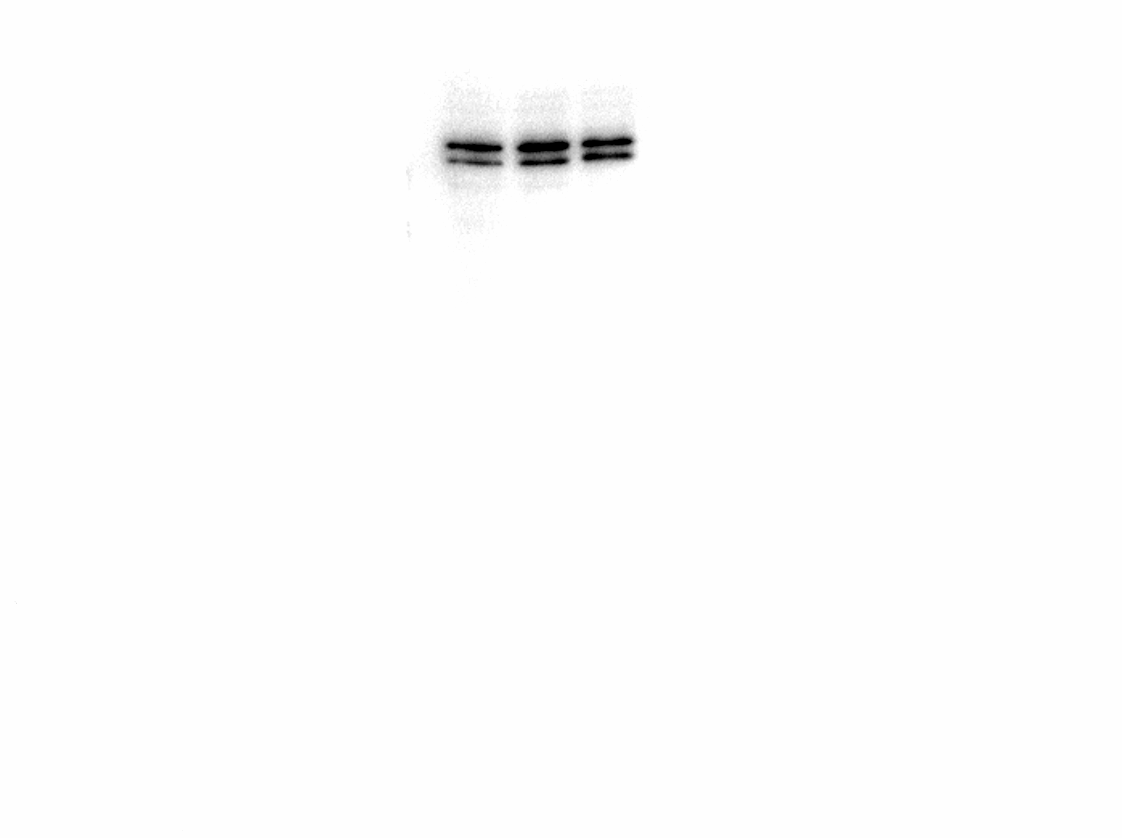

Supplement: Figure 7—source data 1. [file elife-97051-fig7-data1.zip › Figure7B-source data 1. Raw unedited gels for Figure 7B/MAPK.tif]

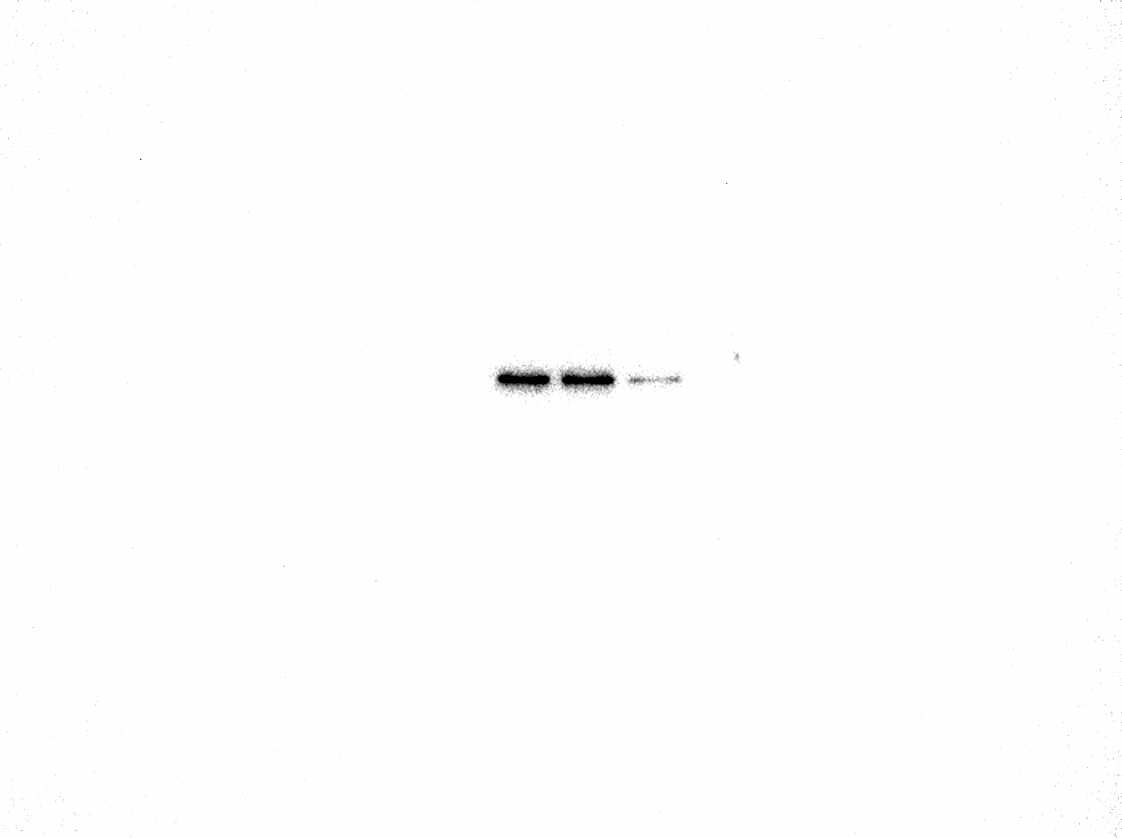

Supplement: Figure 7—source data 1. [file elife-97051-fig7-data1.zip › Figure7B-source data 1. Raw unedited gels for Figure 7B/pAKT.tif]

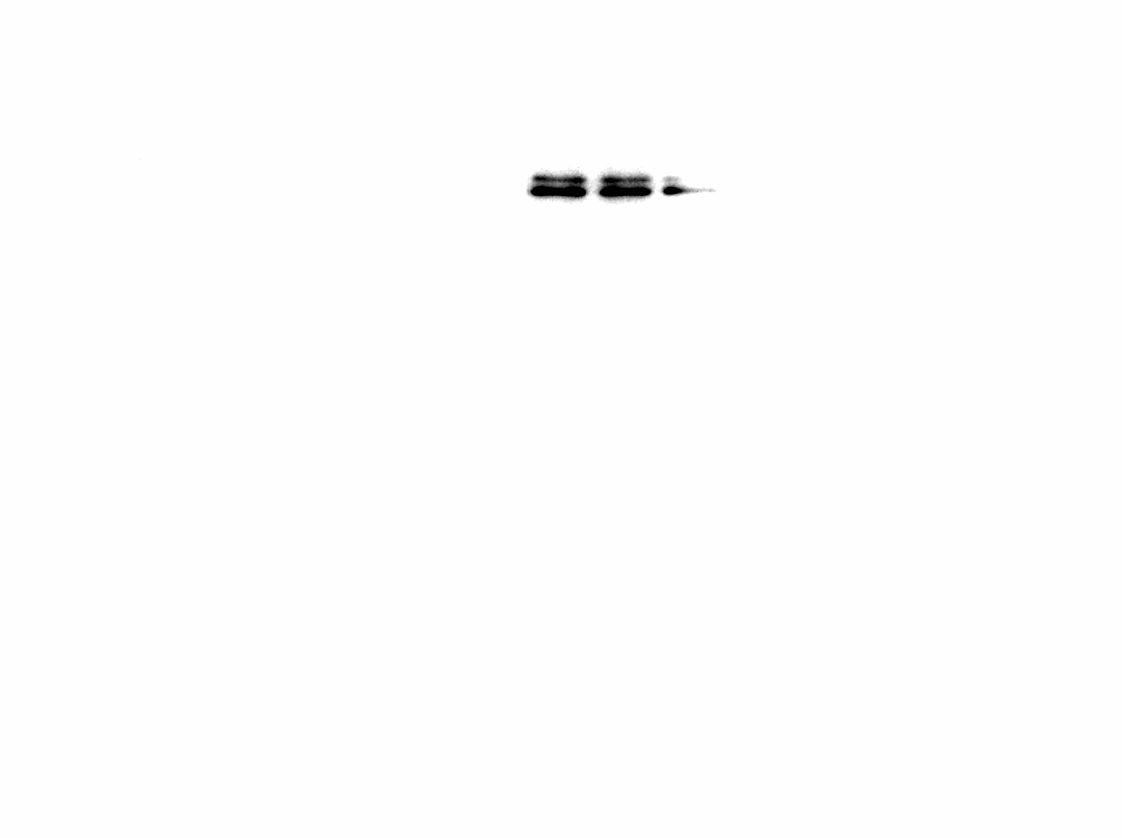

Supplement: Figure 7—source data 1. [file elife-97051-fig7-data1.zip › Figure7B-source data 1. Raw unedited gels for Figure 7B/pMAPK.tif]

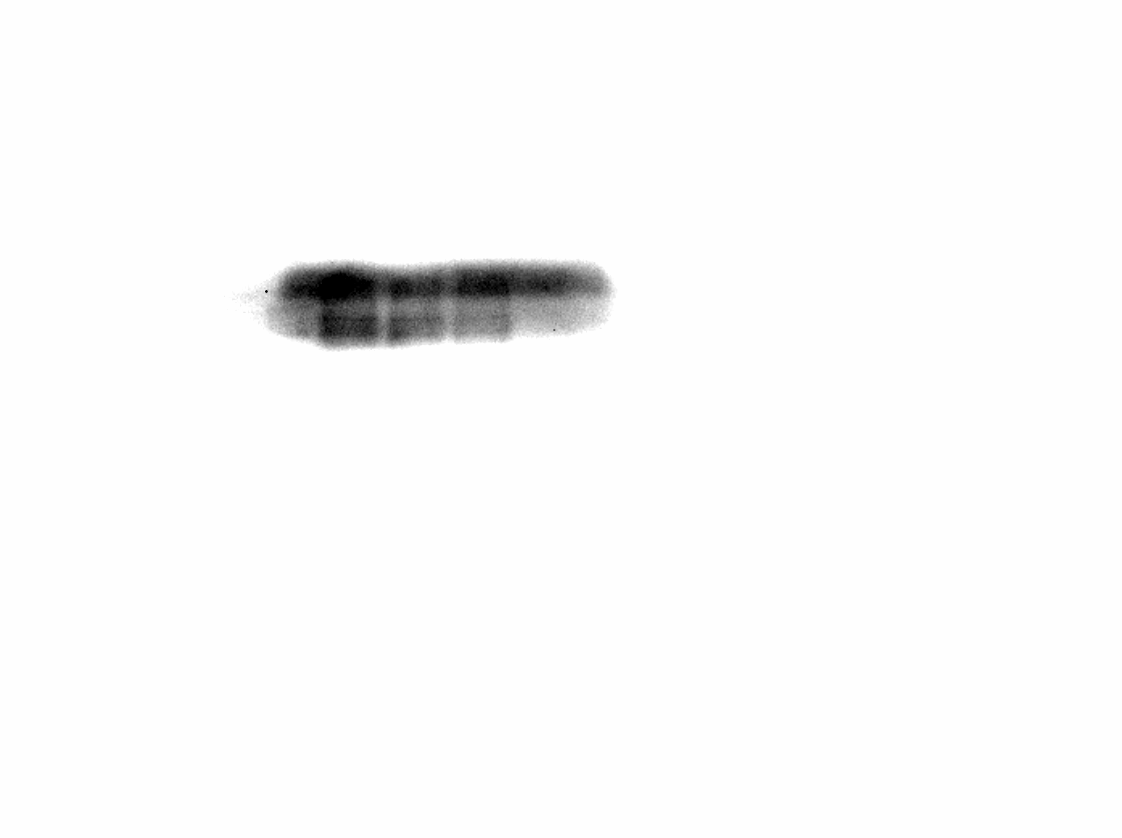

Supplement: Figure 7—source data 1. [file elife-97051-fig7-data1.zip › Figure7B-source data 1. Raw unedited gels for Figure 7B/Vinculin1.tif]

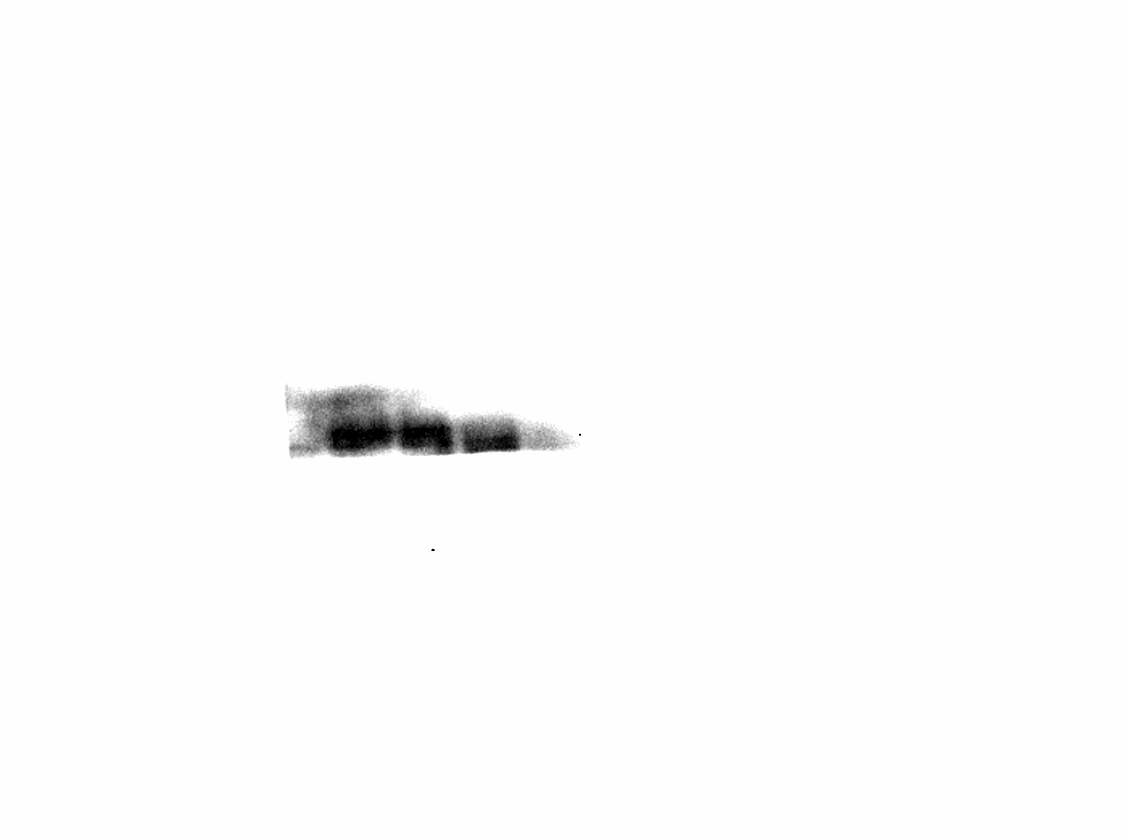

Supplement: Figure 7—source data 1. [file elife-97051-fig7-data1.zip › Figure7B-source data 1. Raw unedited gels for Figure 7B/Vinculin2.tif]

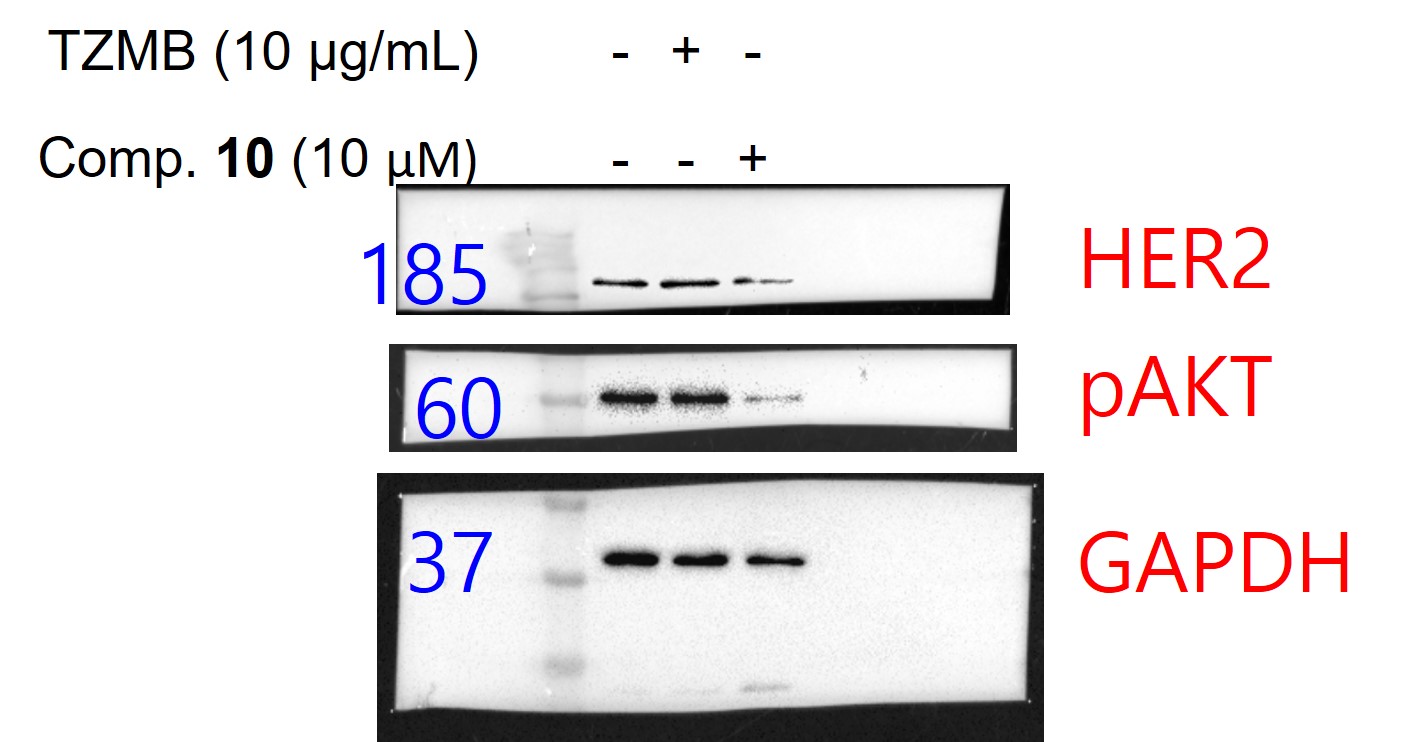

Supplement: Figure 7—source data 2. [file elife-97051-fig7-data2.zip › 1.jpg]

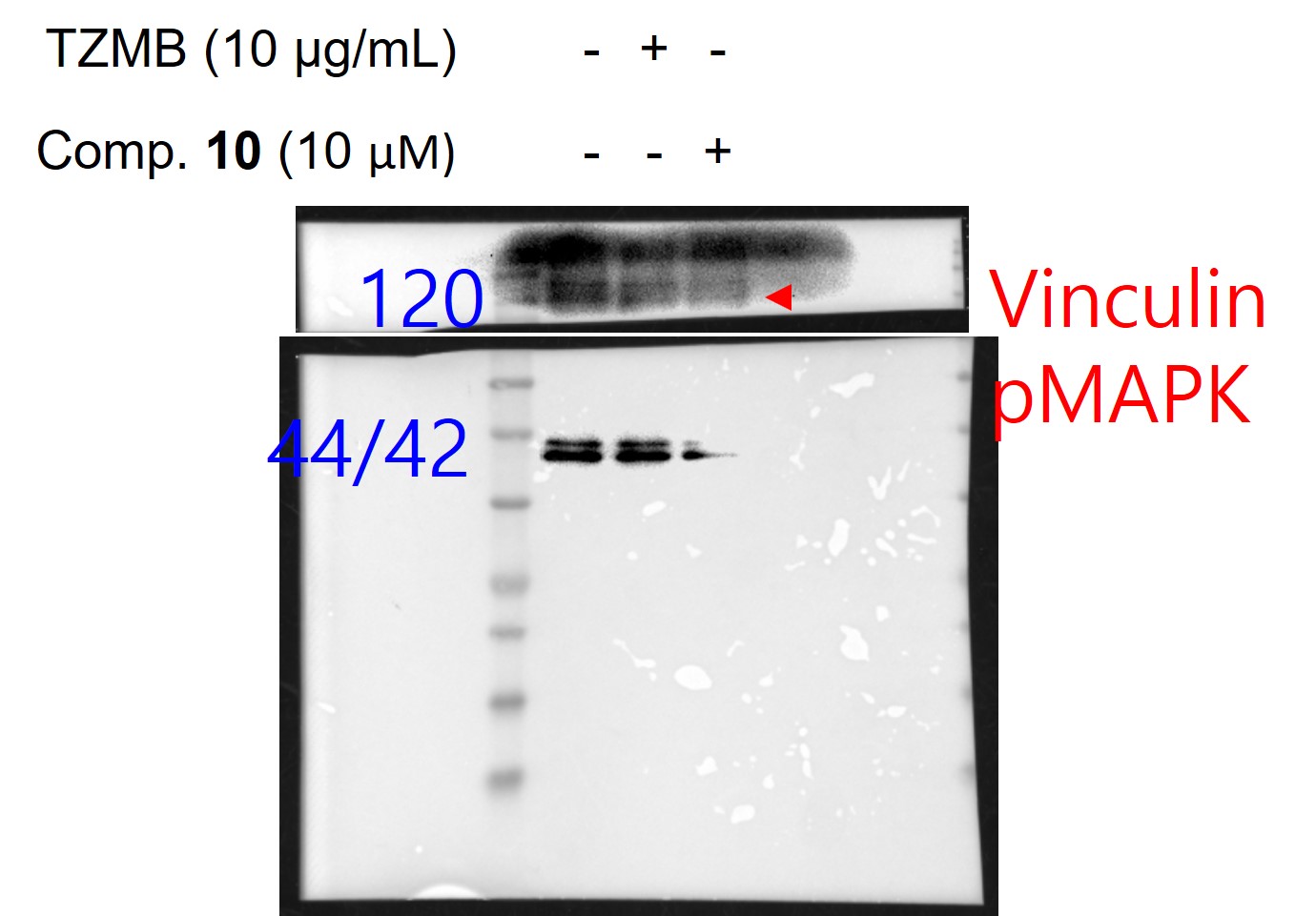

Supplement: Figure 7—source data 2. [file elife-97051-fig7-data2.zip › 2.jpg]

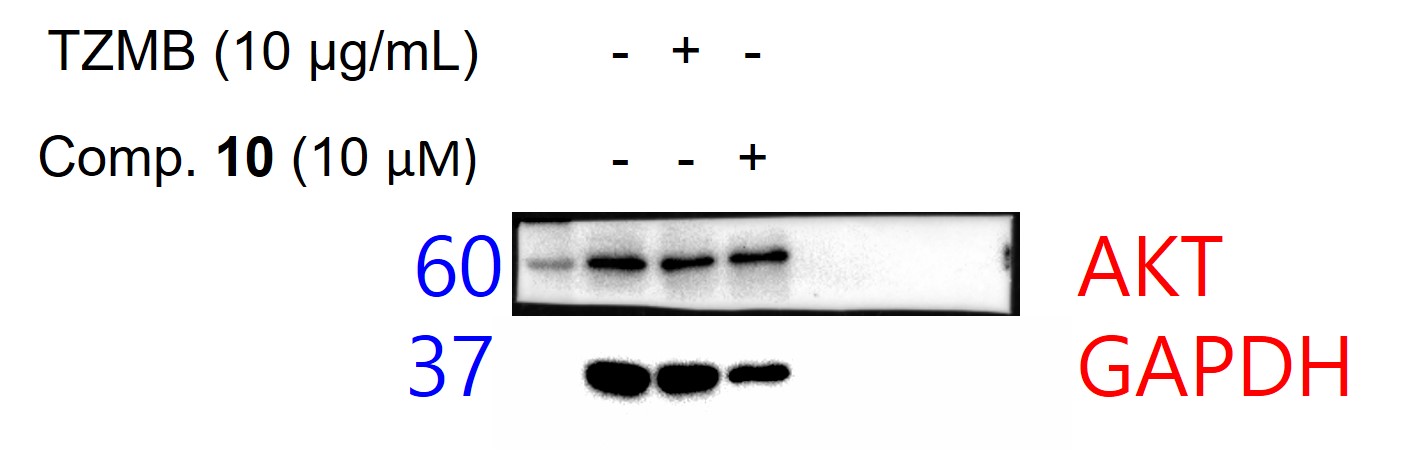

Supplement: Figure 7—source data 2. [file elife-97051-fig7-data2.zip › 3.jpg]

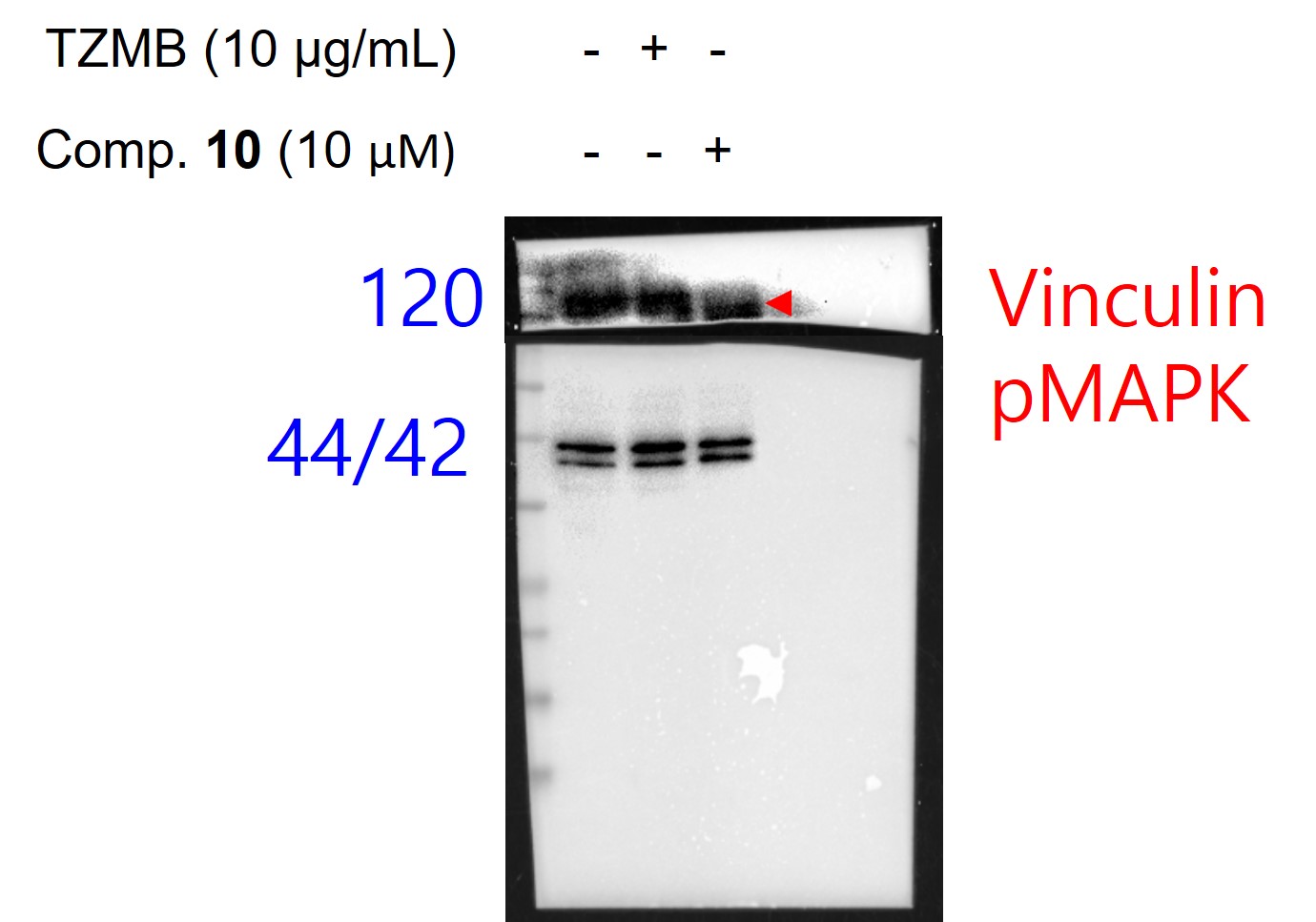

Supplement: Figure 7—source data 2. [file elife-97051-fig7-data2.zip › 4.jpg]

## Source Data 2

### Uncropped blot images of Figure 7B

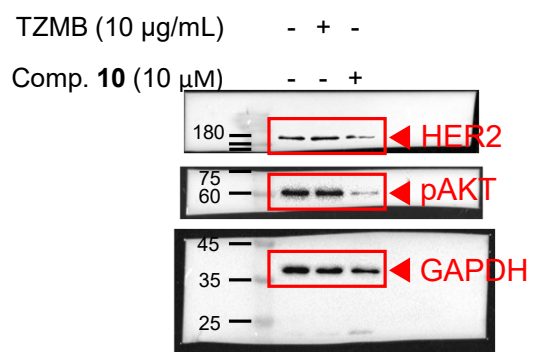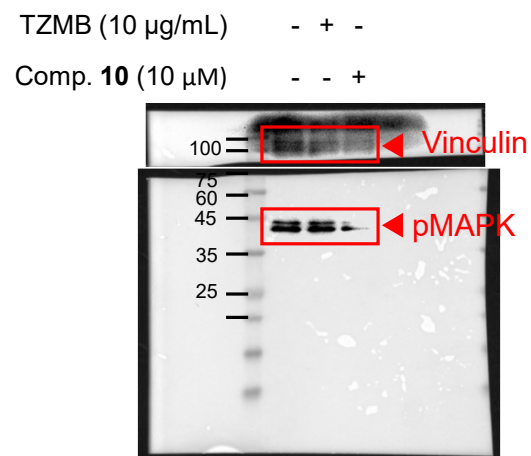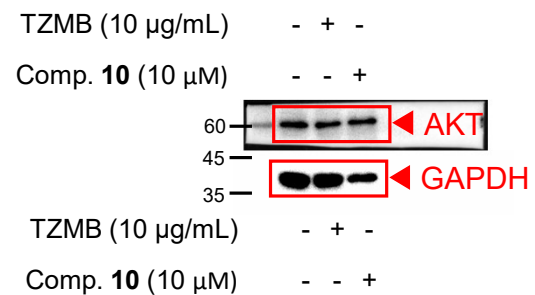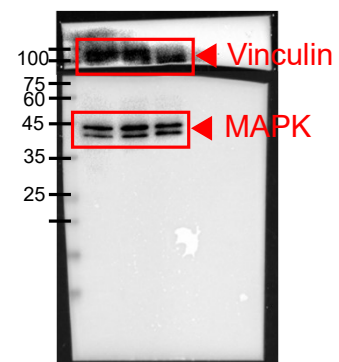

Supplement: Figure 7—source data 2. [file elife-97051-fig7-data2.zip › Source data with molecular weight marker_Part6.pdf]
